# Neural oscillations track recovery of consciousness in acute traumatic brain injury patients

**DOI:** 10.1002/hbm.25725

**Published:** 2022-01-25

**Authors:** Joel Frohlich, Julia S. Crone, Micah A. Johnson, Evan S. Lutkenhoff, Norman M. Spivak, John Dell'Italia, Joerg F. Hipp, Vikesh Shrestha, Jesús E. Ruiz Tejeda, Courtney Real, Paul M. Vespa, Martin M. Monti

**Affiliations:** ^1^ Department of Psychology University of California Los Angeles Los Angeles California USA; ^2^ Vienna Cognitive Science Hub University of Vienna Vienna Austria; ^3^ Department of Neurosurgery UCLA Brain Injury Research Center, David Geffen School of Medicine, University of California Los Angeles Los Angeles California USA; ^4^ Roche Pharma Research and Early Development Roche Innovation Center Basel Basel Switzerland

**Keywords:** biomarker, coma, consciousness, EEG, mesocircuit, neural oscillations, traumatic brain injury

## Abstract

Electroencephalography (EEG), easily deployed at the bedside, is an attractive modality for deriving quantitative biomarkers of prognosis and differential diagnosis in severe brain injury and disorders of consciousness (DOC). Prior work by Schiff has identified four dynamic regimes of progressive recovery of consciousness defined by the presence or absence of thalamically‐driven EEG oscillations. These four predefined categories (ABCD model) relate, on a theoretical level, to thalamocortical integrity and, on an empirical level, to behavioral outcome in patients with cardiac arrest coma etiologies. However, whether this theory‐based stratification of patients might be useful as a diagnostic biomarker in DOC and measurably linked to thalamocortical dysfunction remains unknown. In this work, we relate the reemergence of thalamically‐driven EEG oscillations to behavioral recovery from traumatic brain injury (TBI) in a cohort of *N* = 38 acute patients with moderate‐to‐severe TBI and an average of 1 week of EEG recorded per patient. We analyzed an average of 3.4 hr of EEG per patient, sampled to coincide with 30‐min periods of maximal behavioral arousal. Our work tests and supports the ABCD model, showing that it outperforms a data‐driven clustering approach and may perform equally well compared to a more parsimonious categorization. Additionally, in a subset of patients (*N* = 11), we correlated EEG findings with functional magnetic resonance imaging (fMRI) connectivity between nodes in the mesocircuit—which has been theoretically implicated by Schiff in DOC—and report a trend‐level relationship that warrants further investigation in larger studies.

## Graphical abstract

In this work, we relate the reemergence of thalamically‐driven EEG oscillations to behavioral recovery from traumatic brain injury (TBI) in a cohort of *N* = 38 acute patients with moderate‐to‐severe TBI and an average of 1 week of EEG data recorded per patient. Our work tests and supports a model of recovery by Schiff, showing that it outperforms a data‐driven clustering approach and may perform equally well compared to a more parsimonious categorization. In a subsample of patients (*N* = 11), we correlated EEG findings with fMRI connectivity between nodes in the mesocircuit—which has been theoretically implicated by Schiff in disorders of consciousness (DOC)—and report a trend‐level relationship that warrants further investigation in larger studies.

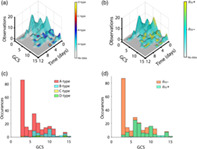

**Abbreviations**
AFNI: analysis of functional neuroimages
BOLD: blood oxygen level dependent
DOC: disorders of consciousness
EDF: European data format
EEG: electroencephalography
FDR: false discovery rate
fMRI: functional magnetic resonance imaging
FWHM: full‐width at half‐maximum
GCS: Glasgow coma scale
GOSe: Glasgow outcome scale extended
GPi: Globus Pallidus interna
ICA: independent component analysis
ICU: intensive care unit
LMM: linear mixed model
MSN: medium spiny neuron
PCA: principal component analysis
PCC: posterior cingular cortex
PFC: prefrontal cortex
PSD: power spectral density
ROI: region of interest
RPI: right‐to‐left posterior‐to‐anterior inferior‐to‐superior
TBI: traumatic brain injury

## 1 INTRODUCTION

Disorders of consciousness (DOC), such as coma and vegetative state, are conditions in which both responsiveness and conscious awareness are diminished as a result of an insult such as brain injury or ischemia (Giacino, Fins, Laureys, & Schiff, 2014). Biomarkers of recovery are greatly needed in DOC to inform prognosis and differential diagnosis, as diagnostic error rates in DOC are as high as 40%, largely owing to the challenges posed by behavioral assessments of level of consciousness (Andrews, Murphy, Munday, & Littlewood, 1996; Childs, Mercer, & Childs, 1993; Schnakers et al., 2009). Diagnostic biomarkers are needed in DOC to identify instances of covert consciousness, that is, consciousness that occurs in the absence of behavioral responsiveness (Huang et al., 2018; Monti et al., 2010). Furthermore, patients' prognoses are also frequently inaccurate, with many families often advised to consider withdraw of care despite over two thirds of patients recovering consciousness when DOC results from traumatic brain injury (TBI) (Giacino et al., 2014; Peberdy et al., 2003; Turgeon et al., 2011). However, predicting which patients are likely to recover consciousness in the absence of prognostic biomarkers remains challenging, thus underscoring the need for such biomarkers (Provencio et al., 2020).

EEG is an attractive modality for biomarkers of postinjury recovery. As a direct readout of cortical activity, EEG is inexpensive, portable, and easily deployed at the bedside. In particular, EEG may be well suited to test hypotheses concerning the role of functional reafferentation of the cortex during coma recovery, that is, restoration of thalamocortical integrity. One such hypothesis, the mesocircuit model, theorizes that the globus pallidus interna (GPi) is disinhibited following diffuse brain injury, and thus, silences the central thalamus, resulting in functional deafferentation of the cortex (Schiff, 2010, 2016). Thus, recovery from DOC requires restoration of striatal functioning and thalamocortical integrity. The latter may be inferred from EEG, given that cortical oscillations such as theta and alpha are thought to be driven by the thalamus (Hughes & Crunelli, 2005; Lindgren et al., 1999; Liu et al., 2012; Sarnthein & Jeanmonod, 2007; Sarnthein, Morel, Von Stein, & Jeanmonod, 2003; Schreckenberger et al., 2004). As such, the loss and recovery of thalamocortical integrity is visible in noninvasive recordings and motivates the “ABCD” model by Schiff.

Based on the assumption that specific cortical oscillations indicate varying levels of thalamocortical integrity, Schiff (2016) has defined four dynamic regimes that build on the mesocircuit model, each detectable with EEG and corresponding to a thalamocortical state that indicates progressive circuit recovery. In particular, this model emphasizes thalamic projections to frontal cortical areas, given the privileged role of central thalamic nuclei in anterior forebrain arousal (Schiff, 2020). These EEG types, labeled A–D (hence, ABCD model) are summarized in Table 1. Later types (C, D) denote more progressive recovery (i.e., are “better”) than earlier types, (A, B), which correspond to a quiescent thalamic state. Specifically, A‐type EEG spectra (featuring no or only low frequency oscillations) are thought to indicate complete cortical deafferentation on a circuit level and a vegetative state on a behavioral level, whereas B‐type spectra (featuring theta oscillations) indicate severe deafferentation and a minimally conscious state. Next, C‐type spectra (featuring theta and beta oscillations) may occur when thalamic nuclei fire in burst mode, corresponding to less severe deafferentation and emergence from the minimally conscious state. Finally, D‐type spectra (featuring alpha and beta oscillations) indicate an approximately normal EEG, corresponding to tonic firing of thalamic nuclei and a normal capacity for wakeful consciousness. During the progression from A to D, excitatory synaptic background activity, as well as metabolic rates in cortical, central thalamic, and pallidal tissues, are increasingly restored to normal levels (Comanducci et al., 2020).

**TABLE 1:** EEG types, criteria, and descriptive statistics

	Theoretical meaning for thalamus	Theta rhythm	Alpha rhythm	Beta rhythm	Proportion of EEG observations	GCS (mean ± *SD*)	GCS (min–max)
A‐type	Quiescent	No	No	No	71.6% (229/320)	5.9 ± 3.1	3–15
B‐type	Nearly quiescent	Yes	No	No	15.0% (48/320)	9.3 ± 2.8	4–15
C‐type	Bursting	Yes	No	Yes	1.88% (6/320)	8.8 ± 4.4	3–14
D‐type	Tonically active	No	Yes	Yes	0.94% (3/320)	9.0 ± 4.4	6–14
θα−	No theory	No	No	N/A	69.1% (221/320)	5.9 ± 3.1	3–15
θα+	No theory	Yes (or alpha)	Yes (or theta)	N/A	30.9% (99/320)	8.0 ± 2.9	3–15

*Note*: Theoretical meanings are based on the original ABCD model by (Schiff, 2016). Some EEG observations (10.6%) could not be classified according to the ABCD model due to peak combinations that did not fit any ABCD type (e.g., a peak in beta without an accompanying peak in theta or alpha).

Two recent studies have tested the mesocircuit hypothesis in the context of the ABCD model using EEG. Forgacs et al. (2017) found that EEGs from 44 patients who had lost consciousness following cardiac arrest displayed a progression of EEG patterns consistent with the ABCD model. In particular, EEG patterns indicative of greater circuit‐level recovery correlated with better outcomes at hospital discharge. More recently, Alkhachroum et al. (2020) used the ABCD model to examine recovery in DOC patients with largely anoxic etiologies treated with amantadine. The best recorded ABCD type increased (A–D) linearly with the percentage of patients who recovered the ability to follow commands.

The foregoing studies offer first evidence for the ABCD model. However, it is unknown how EEG dynamic regimes relate empirically to recovery of the mesocircuit in DOC and whether these EEG types can be used as biomarkers. Deploying the ABCD model as a clinical biomarker will depend crucially on whether the different types can be detected at an acute stage and related to recovery of the mesocircuit. With the exception of three patients (Alkhachroum et al., 2020), the ABCD model has never been applied to patients with severe TBI in the acute stage.

Moreover, prior studies have used manual scoring to categorize ABCD type (Alkhachroum et al., 2020; Forgacs et al., 2017), which is inefficient in clinical application; thus, it is necessary to test whether similar results can be achieved using automated methods based on quantitative EEG criteria. Finally, a weakness of the ABCD model is that not all EEGs can be classified into one of four types, nor will one type always capture a majority of EEG channels. Alternative models, for which EEGs are always classifiable and one of two types always captures a majority of channels, warrant investigation. It remains unknown how the ABCD model compares to other approaches, including more parsimonious models, models based on spectral power rather than peaks, and data‐driven clustering.

To address these open questions, we used objective criteria categorization of acute EEG following moderate‐to‐severe TBI to test the ABCD model in a cohort of 38 severe TBI patients in the intensive care unit (ICU). Our study tested four hypotheses: ABCD type relates to (a) acute and (b) chronic (> 5 months) behavioral recovery in patients, (c) the ABCD model outperforms alternative EEG categorizations that are more parsimonious, use different features, or are data‐driven, and (d) the reemergence of neural oscillations tracks mesocircuit recovery. Our results show that the reemergence of neural oscillations >4 Hz tracks recovery of behavioral responsiveness and consciousness in acute patients following severe TBI and, moreover, that EEG categories defined by spectral peaks outperform a data‐driven clustering approach. We also found that spectral power generally either did not predict these same variables or failed to improve peak‐based model fits, with the exceptions of relative alpha (8–12 Hz) power and absolute beta (12–35 Hz) power. Finally, we also identified promising relationships between EEG and mesocircuit recovery in a subset of 11 patients.

## 2 MATERIALS AND METHODS

### 2.1 Data collection

We collected continuous recordings over several days from each patient in our cohort. This strategy utilized a vast amount of data (range: 15.0–320 hr per patient, mean ± *SD*: 163 ± 62.5 h) in a sample of 41 patients admitted at the Ronald Reagan UCLA Medical Center Neuroscience/Trauma ICU from December 2015 to February 2020 following moderate‐to‐severe TBI. The sample size was determined by patient availability and was not set a priori. Our inclusion criteria were as follows: patients were retained if they demonstrated an admission Glasgow Coma Scale (GCS; Teasdale & Jennett, 1974) score ≤8 or an admission GCS score of 9–14 with computed tomography (CT) evidence of intracranial bleeding. Our exclusion criteria eliminated patients with any of the following: GCS > 14 with nonsignificant head CT, history of neurologic disease or TBI, and brain death. Ethics approval for the study was obtained by the UCLA IRB. Families of patients gave consent to participate in the study in accordance with the Declaration of Helsinki.

**FIGURE 1:**
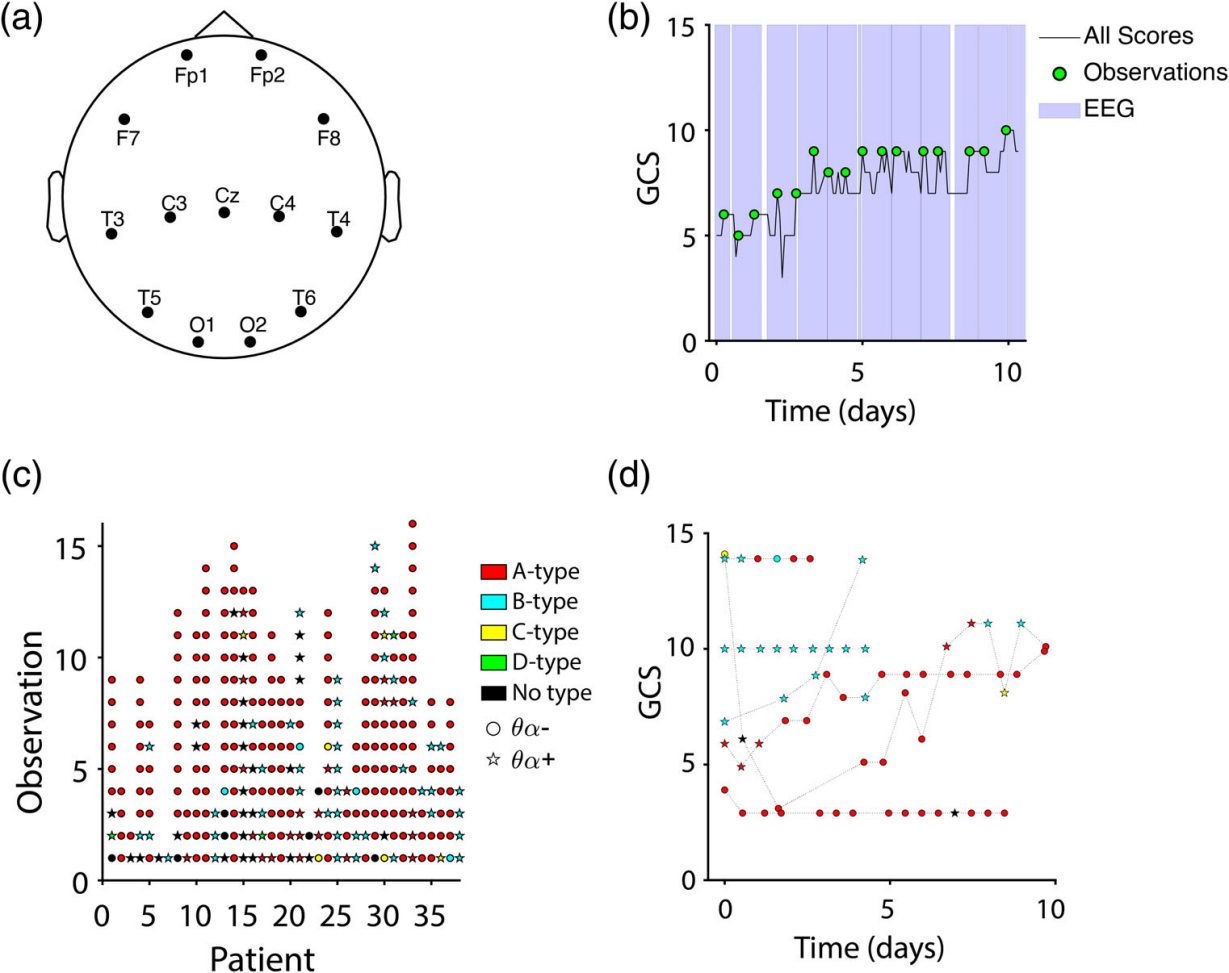
EEG types and behavioral trajectories. (a) Thirteen EEG channels common to all patients were imported for analysis. Actual channel positions varied from patient to patient to accommodate bone flaps and injuries. (b) EEG observations (green circles) were sampled at timepoints corresponding to local maxima of the Glasgow Coma Scale (GCS, black trace), with a 12‐hr buffer in between observations. Purple highlights show times with available EEG data. Time is referenced to the patient's earliest GCS score. (c) ABCD type (color) and θα type (shape) for all patients and observations. Successive observations are spaced equally regardless of chronical time elapsed. (d) Example GCS trajectories for six representative patients showing ABCD type (color) and θα type (shape) at each observation along the trajectory. Time is referenced to earliest time with EEG for each patient

While patients were in the ICU, clinical EEG was recorded continuously for multiple days using a sparse montage with 13–17 channels (Cz reference). Channel placement was modified from standard positions to accommodate bone flaps and injury sites in individual patients. Given the small number of channels and variable channel placement, we did not perform source localization or surface Laplacian montages in our analyses (Cohen, 2014). EEG data were acquired from most patients using a Nicolet Monitor (Natus Medical, Inc., Pleasanton, CA); however, data from two patients (#7 and #26, see Table 1) were acquired using systems by Moberg ICU Solutions (Moberg Research, Inc., Ambler, PA). Data were de‐identified and exported as European data format (EDF) files using Persyst software (Persyst Development Corporation, Solana Beach, CA). Behavioral assessments were performed several times daily using the GCS (i.e., each EEG recording temporally overlapped with data from many GCS assessments of the patient). To analyze patients at peak arousal, we extracted 30‐min EEG observations from 13 channels common to all patients (Figure 1a) from timepoints corresponding to high GCS scores, with EEG observations spaced a minimum of 12 hr apart. This was accomplished by sorting each patient's GCS scores that overlapped with EEG recordings from high to low, appending the highest score to a second list, and then crawling down the first list of GCS scores to find the next timepoint that was at least 12 hr apart from any timepoint on the second list and then adding this timepoint to the second list, and so on until no additional timepoints could be added to the second list without violating the 12‐hr buffer. EEG observations (30 min each) were then extracted according to the second list's timepoints (Figure 1b). Finally, as a chronic outcome measure, the Glasgow Outcome Scale Extended (GOSe, Jennett, Snoek, Bond, & Brooks, 1981) was administered approximately 6 months (190 ± 33 days, mean ± *SD*; min = 158 days, max = 318 days) postinjury either in‐person or by phone to patients and/or family members.

Additionally, we acquired functional magnetic resonance imaging (fMRI) data from patients at the earliest date when patients were stable enough to be scanned safely with an echo‐planar imaging (EPI) sequence on a 3 Tesla Siemens TimTrio (Siemens AG, Munich, Germany) MRI machine at the UCLA Ronald Reagan Medical Center. Anatomical data were acquired with a magnetization prepared rapid gradient echo imaging sequence (MPRAGE, TR = 1,900 ms, TE = 3.52 ms, FA = 98); other MRI sequences not used in this study were also acquired in the same session. Because scanning did not always coincide with EEG acquisition, only a subset of patients had fMRI data acquired within 48 hr of at least one EEG observation (see Table 2).

**TABLE 2:** Patient details and demographics

IDs	Age at injury (years)	Sex	Number of usable EEG observations	Mean GCS	GOSe	Time to follow up (days)	Included in fMRI analysis?	Comments
1	19	Male	9	10	7	181	No	
2	20	Female	4	4	3	192	Yes	
3	20	Male	2	7	6	170	No	Excluded from LMMs due to insufficient number of observations
4	22	Male	9	7	6	179	No	EEG recorded with Moberg ICU solutions
5	22	Male	7	6	3	164	No	
6	22	Male	1	10	4	158	Yes	Excluded from LMMs due to insufficient number of observations
7	22	Male	1	13	8	184	No	Excluded from LMMs due to insufficient number of observations
8	23	Female	12	7	7	182	Yes	Excluded from non‐fMRI analyses due to missing medication data
9	23	Female	3	7	3	183	Yes	
10	25	Male	11	3	8	180	Yes	
11	26	Male	14	3	2	161	No	
12	27	Male	3	10	7	179	No	
13	28	Male	13	8	4	177	No	
14	31	Male	15	3	3	246	Yes	
15	31	Male	13	7	6	182	No	
16	31	Male	13	4	3	183	No	
17	32	Male	8	6	3	177	No	
18	33	Male	11	6	N/A	N/A	Yes	Excluded from prediction of chronic outcome (missing GOSe score)
19	36	Female	9	6	7	188	No	
20	37	Male	8	4	2	177	No	
21	39	Male	12	7	3	179	Yes	
22	40	Male	2	10	5	173	No	Excluded from LMMs due to insufficient number of observations
23	43	Male	4	7	5	203	No	
24	44	Male	12	11	2	172	No	
25	45	Male	9	10	3	182	No	
26	47	Male	4	8	5	184	No	EEG recorded with Moberg ICU solutions
27	49	Male	6	14	6	174	No	
28	54	Male	9	8	5	189	No	
29	55	Male	15	7	N/A	N/A	No	Excluded from prediction of chronic outcome (missing GOSe score)
30	55	Female	13	14	N/A	N/A	Yes	Excluded from prediction of chronic outcome (missing GOSe score)
31	57	Male	11	7	3	224	Yes	
32	58	Female	11	9	1	N/A	No	Patient deceased
33	59	Male	16	6	3	279	Yes	
34	61	Male	4	8	7	318	No	
35	62	Female	8	10	3	181	No	
36	72	Male	6	14	6	182	No	
37	75	Male	8	7	N/A	N/A	No	Excluded from prediction of chronic outcome (missing GOSe score)
38	84	Male	4	7	5	177	No	

*Note*: Our heavily male sample reflects higher risk for TBI in males. “Time to follow up” gives the number of days postinjury when GOSe assessments were performed. Note that GOSe = 1 indicates that the patient was deceased (thus, for Patient 32, the time to follow up was not applicable). Comments give additional details including which analyses, if any, patients were excluded from and why.

Abbreviation: N/A, not applicable.

### 2.2 EEG preprocessing and analysis

A total of 419 EEG observations from 41 patients entered preprocessing. After importing EDF files to MATLAB (version R2019b, The MathWorks, Inc., Natick, MA) for analysis, all datasets were re‐referenced to average and bandpass filtered 0.5–45 Hz (finite impulse response filter, filter order: 2× sampling rate). Sections of data containing gross physiological or technical artifacts were manually free‐selected and excluded from analysis. Independent component analysis (ICA) was used to remove stereotyped artifacts such as muscle activity (algorithm: FastICA, Hyvarinen, 1999; Jung et al., 2000) Because channel locations were highly variable (see above) precluding spine interpolations of noisy channels, we also used ICA to remove noisy channel components. During quality control, EEG observations were eliminated due either to (a) persistent physiological and/or technical artifacts not removable with ICA or (b) insufficient data length (defined as <30 valid 1 Hz frequency transform windows). EEG recordings were heavily contaminated with technical artifacts or corrupted from three patients, who were excluded from further analysis. Following preprocessing and artifact reduction, we implemented a frequency transform using log‐spaced Morlet wavelets (1–45 Hz, 8 wavelets per octave; *f/σ*_*f*_ = 8.7; *σ*_*f*_, spectral SD); see Supporting Information Materials and Methods for details.

### 2.3 EEG classification

To classify EEG observations according to ABCD type, we spline‐interpolated channel‐level power spectral densities (PSDs) to achieve 100 frequency bins per octave. Next, we identified local maxima in PSDs [MATLAB function: findpeaks, min width = 0.1 log_2_(Hz), min prominence = 0.001 log_10_(*μ*V^2^/Hz)] and determined the ABCD type using criteria in Table 1. EEG frequency bands were defined as follows (lower bounds are exclusive and upper bounds are inclusive): delta, 1–4 Hz; theta, 4–8 Hz; alpha, 8–12 Hz; beta, 12–35 Hz. For each EEG observation, we used locations of local maxima of PSDs to determine which ABCD type, if any, each channel belonged to. We then took the mode across all classifiable channels to determine the type of the EEG; ties were broken using the most progressive type (e.g., B‐type in the event of an equal number of channels in the A‐type and the B‐type). EEG observations were only considered unclassifiable if all channels were unclassifiable. We also classified EEG observations according to whether they contained a theta and/or alpha peak (henceforth: θα type) using the mode across channels, given that the long oscillatory periods (>83 ms) of these EEG rhythms are compatible with physiological conduction delays between thalamus and cortex (Swadlow & Waxman, 2012; i.e., thalamocortical communication occurs fully within the excitable phase of the oscillation or one half of the oscillatory period [Fries, 2005]) and are thus especially valuable for inferring thalamocortical integrity.

### 2.4 fMRI data processing

Patients in our cohort are prone to high motion so we have implemented several preprocessing measures and exclusion criteria to cope with motion‐related artifacts (See Supporting Information Materials and Methods). Other details of the preprocessing, including skull‐stripping, segmentation, motion correction, and registration, can also be found in Supporting Information Materials and Methods.

#### 2.4.1 Brain parcellation using independent component analysis

We performed ICA using the GIFT toolbox (http://mialab.mrn.org/software/gift/index.html) to parcellate the brain as implemented in Allen et al. (2014). We chose this parcellation approach (Crone et al., 2015; Crone, Lutkenhoff, Vespa, & Monti, 2020) as opposed to standard atlases, because the brains we investigated were severely injured. Thus, it is problematic to assume that parcellation resulting from the average of young and healthy brains adequately represents function in a brain that has been subject to reorganization due to TBI. We defined the cortical regions of interest (ROIs) at an individual level based on the individual functional covariance using groupICA. See Supporting Information Materials and Methods for further details of the parcellation and seed‐based connectivity analysis.

### 2.5 Principal components space clustering

Having classified EEG observations according to the presence or absence of power spectral peaks, we next asked how the foregoing approach would compare with a data‐driven approach. To implement such an approach, we began by averaging power (unnormalized) across channels for each patient and log‐scaling. Due to the large number of frequency bins (44), we next applied PCA to this feature space and retained the top two PCs according to variance explained. We then used k‐means clustering to identify two clusters in PC space. Only one EEG observation was used per patient for clustering; EEG observations were selected according to the procedure described below under statistical analysis.

### 2.6 Patient medications

Patients in our study were administered a very large number of medications in the ICU. To appropriately account for medications, we first categorized medications from each EEG observation into one of five classes: propofol, opioids, benzodiazepines, barbiturates, and dissociatives (i.e., ketamine and dexmedetomidine). Next, we applied logistic PCA to the resultant matrix of observations × medication classes to reduce the medication data to a two‐dimensional space; note that multiple observations per patient were included. This technique is a variant of PCA that is appropriate for binary variables (Landgraf, 2016; Landgraf & Lee, 2020). See Supporting Information Materials and Methods for further details.

### 2.7 Statistical analysis

To determine the extent to which EEG spectral peaks predicted patients' behavior, we related acute, longitudinal EEG data to both acute, longitudinal behavioral data (i.e., daily maximum GCS scores) and a single, chronic behavioral datum (i.e., chronic GOSe score). For the former, we used linear mixed models (LMMs) with random intercepts, that is, varying‐intercept models. We allowed intercepts but not slopes to vary between patients as we expected patients to have different baselines but did not expect predictors to exert differing levels of influence across patients. Furthermore, given that all models had at least five predictor variables, modeling random slopes for each predictor would result in a cumbersome level of model complexity relative to our sample size. This LMM had the formula

(1) $$\mathrm{GCS}∼1+\mathrm{EEG}+\mathrm{AGE}+\mathrm{SEX}+{\mathrm{PC}}_{1}+{\mathrm{PC}}_{2}+\left(1|\text{PATIENT}\right)$$

where GCS is the daily maximum GCS score, EEG is either an ordinal variable [A‐type, B‐type, C‐type, D‐type] or binary variable [A‐type, non‐A‐type; or θα−, θα+], AGE is the age at injury in years rounded down to the nearest whole number, SEX is the sex of the patient, and PC_1_ and PC_2_ are the first and second PCs yielded by the logistic PCA of medications. LMMs were fit in MATLAB using the function fitlme. Instances of unclassifiable ABCD type were treated as missing data. Patients with fewer than three usable EEG observations were excluded from this analysis due to an insufficient number of observations for inclusion in LMMs.

Additionally, we utilized GCS subscales to infer consciousness in patients and related the same predictors to a binary variable denoting conscious state. Specifically, we inferred the presence of consciousness from a GCS motor score ≥5 or a GCS verbal score ≥4 (see Crone et al., 2020 for further details). We then tested the relationship between predictors and conscious state using generalized LMMs (GLMMs; MATLAB function fitglme) with the logit link function and the formula:

(2) $$\text{CONSCIOUS∼}1+\mathrm{EEG}+\mathrm{AGE}+\mathrm{SEX}+{\mathrm{PC}}_{1}+{\mathrm{PC}}_{2}+\left(1|\text{PATIENT}\right)$$

where CONSCIOUS is a binary variable denoting the presence or absence of consciousness. For each of the above LMMs and GLMMs, we performed an *F*‐test to evaluate the EEG term.

An alternative model that predicts GCS scores and conscious state based on EEG spectral band power, rather than peak combinations, was also assessed. Separate models were fit for the absolute spectral power (unnormalized) and relative power (normalized by the total 1–45 Hz power). Specifically, we fit LMMs with the formula

(3) $$\mathrm{GCS}∼1+\text{DELTA}+\text{THETA}+\text{ALPHA}+\text{BETA}+\mathrm{AGE}+\mathrm{SEX}+\text{PC1}+\text{PC2}+\left(1|\text{PATIENT}\right)$$

where the following variables are log‐scaled absolute or relative integrated power (lower bounds are exclusive, upper bounds are inclusive): DELTA, 1–4 Hz; THETA, 4–8 HZ; ALPHA, 8–12 Hz; BETA, 12–35 Hz. We did not use gamma power as a predictor for two reasons: (a) gamma power is confounded by difficult to remove muscle artifacts that are likely to be correlated with GCS scores, and (b) because we used logarithmically spaced Morlet wavelets, there were only two frequency bins in the gamma range between 35 Hz and the lowpass filter cutoff frequency at 45 Hz. Next, we used GLMMs with the logit link function to predict conscious state with the formula

(4) $$\text{CONSCIOUS}∼1+\text{DELTA}+\text{THETA}+\text{ALPHA}+\text{BETA}+\mathrm{AGE}+\mathrm{SEX}+\text{PC1}+\text{PC2}+\left(1|\text{PATIENT}\right)$$

For the second aim (i.e., relating multiple longitudinal EEG observations per patient to a single chronic outcome), we were unable to fit LMMs or GLMMs because they are not compatible with an unbalanced design featuring dynamic/longitudinal predictors with a static outcome variable. Rather than arbitrarily choosing one EEG timepoint for each patient to create a balanced design, we utilized multiple linear regression models with a resampling approach that randomly sampled one EEG observation per patient with replacement for each of 9,999 resamples and constructed an empirical distribution of test statistics. Resamples that yielded invalid combinations (i.e., a rank deficient regression design matrix) were discarded and replaced with a new sampling. We reported the results of the resample that yielded the median *t*‐statistic for the variables EEG, DELTA, THETA, ALPHA, or BETA; for this reason, the number of resamples N was chosen as an odd number such that the middle‐most test statistic would be defined. Our initial multiple linear regression model was specified as

(5) $$\text{GOSe}=\beta_{0}+\beta_{1}\text{EEG}+\beta_{2}\text{AGE}+\beta_{\text{3}}\mathrm{SEX}+\beta_{4}\text{PC1}+\beta_{5}\text{PC2}+\varepsilon$$

where GOSe is the chronic GOSe score. For alternative models using spectral power as predictors, we used multiple linear regression models with the formula

(6) $$\;\text{GOSe}=\beta_{0}+\beta_{1}\text{DELTA}+\beta_{2}\text{THETA}+\beta_{3}\text{ALPHA}+\beta_{4}\text{BETA}+\beta_{5}\text{AGE}+\beta_{\text{6}}\mathrm{SEX}+\beta_{7}\text{PC1}+\beta_{8}\text{PC2}+\varepsilon$$

Next, we compared GCS and GOSe scores between clusters identified using k‐means clustering. This clustering approach requires only one EEG observation per patient. Because including all data from each patient in our clustering would have biased clusters toward patients with more observations, we once again utilized a resampling approach with replacement using 9,999 resamples to include only one EEG observation per patient per resample. Invalid resamples were discarded and replaced as described above. For each random resampling, we constructed a feature space using channel‐averaged spectral power across all frequency bins, applied PCA, retained the two PCs that accounted for the highest proportion of variance, and identified two clusters in PC space using k‐means clustering. For consistent labeling of clusters across resamples, we used one label for all clusters that had a centroid with a PC_1_‐coordinate ≥0 and another label for those with a PC_1_‐coordinate <0. We then performed multiple linear regression using the model

(7) $$\text{SCORE}=\beta_{0}+\beta_{1}\text{CLUSTER}+\beta_{2}\text{AGE}+\beta_{\text{3}}\mathrm{SEX}+\beta_{4}\text{PC1}+\beta_{5}\text{PC2}+\varepsilon$$

where SCORE is the behavioral measure (GCS, conscious state, or GOSe) and CLUSTER is a binary variable denoting the patient's power space cluster membership. For predicting conscious state (a binary variable), we utilized logistic multiple regression. GCS scores were selected corresponding to the day/time of each patient's randomly resampled EEG observation. The resample yielding the median t‐statistic for CLUSTER was then selected for reporting. Note that this resampling procedure was performed separately for GCS and GOSe.

Finally, to relate EEG to fMRI connectivity, we correlated the mean proportion of channels with a θα peak with the *z*‐scored fMRI connectivity for each of four fMRI ROI pairings: thalamus‐striatum, striatum‐globus pallidus, thalamus‐prefrontal cortex (PFC), and thalamus‐posterior cingulate cortex (PCC). We used θα type rather than ABCD type due to the greater variance and weaker skew of the former (see Section 3). Furthermore, to reduce the number of data points at floor (i.e., all θα−) or ceiling (i.e., all θα+), we choose to examine the proportion of EEG channels with a θα peak, rather than the modal (and thus binary yes/no) θα type of each EEG observation. To derive noise‐robust estimates for each patient, we averaged this proportion across all EEG observations within 48 hr of fMRI acquisition. Correlations were derived using the Pearson coefficient. We did not include covariates in this analysis for two reasons: (a) EEG observations from both before and after fMRI acquisition were included, and so temporal precedence of predictors could not always be established, and (b) because EEG and fMRI are both intimately related measures of brain activity, their relationship is not confounded by the same variables that were covaried for in other analyses. Outliers were identified and excluded from correlation analysis using a threshold of three scaled median absolute deviations from the median of either variable (peak proportion or fMRI connectivity) using the MATLAB function isoutlier with default parameters. To account for multiple testing, we applied false discover rates (FDR, Benjamini‐Hochberg) to correct *p*‐values (Benjamini & Hochberg, 1995).

In all models, we corrected for the four hypothesis tests outlined in the introduction using a Bonferroni correction, yielding a test‐wise criterion of α = .0125 (for fMRI connectivity, this was performed in addition to the FDR correction).

## 3 RESULTS

Following preprocessing and quality control, we retained 320 EEG observations across 38 patients (31 male) with ages ranging from 19 to 84 years (40 ± 17 years, mean ± *SD*). Patient demographics are summarized in Table 2 and Figure S1. The number of usable EEG observations per patient ranged from 1 to 16 (8.4 ± 4.3, mean ± *SD*), with 1.8–30 min of usable data per observation (24 ± 7.2, mean ± *SD*). Thus, despite our modest patient sample size, we analyzed an average of 3.4 hr of data per patient.

EEG observations were associated with medications as follows: opioids (75%), propofol (43%), benzodiazepines (38%), dissociatives (28%), and barbiturates (16%). Following logistic PCA of medication variables, two PCs explained the majority of variance in the presence/absence of medications (66.9%). Each PC exhibited a positive weight for only one medication variable: barbiturates (PC_1_) and dissociatives (PC_2_) (Figure S2). PC_1_ and PC_2_ also featured strong negative weights for propofol and benzodiazepines, respectively (Figure S2).

**FIGURE 2:**
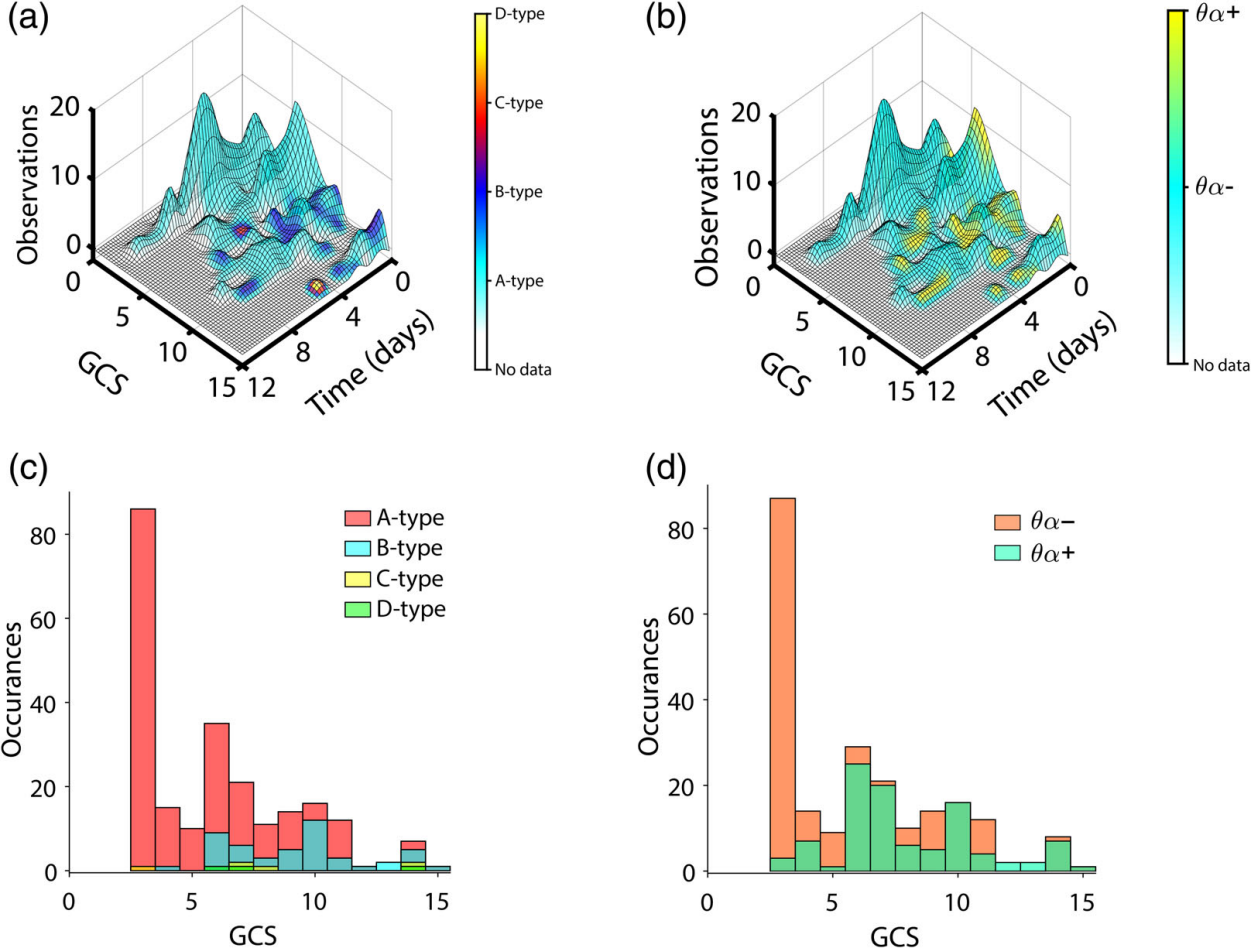
Surface plots and histograms of EEG types. Time is referenced to the earliest time with EEG for each patient (a,b). (a) Number of EEG observations (height of surface plot) and ABCD type (color) across all patients as a function of time and GCS score. Note that low GCS trajectories do not evolve beyond A‐type. (b) Number of EEG observations (height of surface plot) and θα type (color) across all patients as a function of time and GCS score. Note that low GCS trajectories do not evolve beyond θα−. (c) Histogram of GCS scores across all patients' EEG observations color‐coded by ABCD type. (d) Histogram of GCS scores across all patients' EEG observations color‐coded by θα type

**FIGURE 3:**
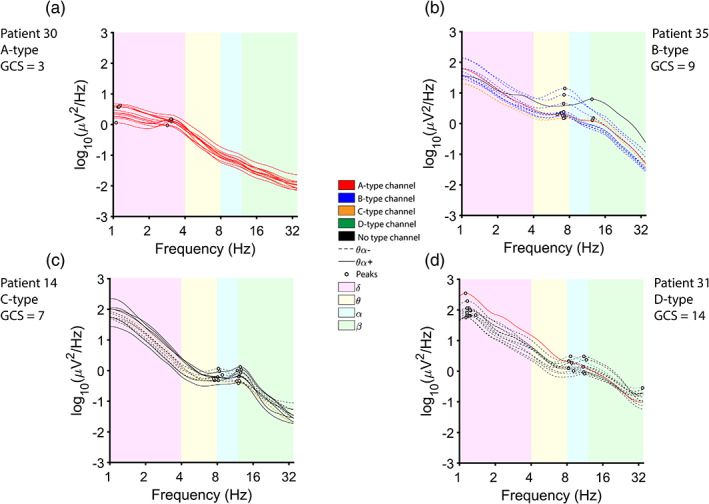
Examples of ABCD model types. Power spectral densities (PSDs) from each channel are color‐coded according to their ABCD type; unclassifiable channels are colored black. Peaks detected for classification are indicated with circles. Channels with a θα peak are dashed. Shaded areas are colored according to frequency band. (a) A‐type EEG corresponding to a GCS score of 3. All channels were categorized as A‐type, and peaks were only present in the delta band. (b) B‐type EEG corresponding to a GCS score of 9. In total, one channel was categorized as A‐type, nine channels as B‐type, two channels as C‐type, and one channel as unclassifiable (due to the presence of a beta peak without an accompanying theta or alpha peak). Eleven channels showed a θα peak. (c) C‐type EEG corresponding to a GCS score of 7. In total, two channels were categorized as C‐type, and the remaining nine channels were uncategorizable. Seven channels showed θα peaks. (d) D‐type EEG corresponding to a GCS score of 14. In total, one channel was categorized as A‐type, one channel as D‐type, and the remaining nine channels were uncategorizable. Because D‐type is a more progressive type than A‐type, the tie between A‐type and D‐type (one channel each) is broken by D‐type. Twelve channels showed a θα peak

EEG observations were most commonly categorized as A‐type (71.6%), with those remaining categorized as B‐type (15.0%), C‐type (1.88%), D‐type (0.94%), or unclassifiable (10.6%). Separately, 30.9% of observations exhibited a peak in the theta‐alpha band in the majority of channels (θα+). Distributions of GCS scores for each type are described in Table 1 and Figure 2. See Figure 3 for examples of each ABCD type and Figure S3 for behavioral trajectories and EEG variables for all patients.

### 3.1 Thalamically‐driven oscillations track behavioral recovery

Given the very small proportion of EEG observations that were categorized as C‐type or D‐type, we opted to create a binary variable by grouping types B, C, and D together, thus avoiding outlier effects. To investigate ABCD type in relation to acute behavioral state, we used an LMM with GCS as the dependent variable. We excluded four patients from the model due to having fewer than three observations, plus an additional patient with missing medication data, yielding n = 33. We found that ABCD type (i.e., A vs. B, C, D) significantly predicted GCS (*p* < 0.0001, *F*_(1,268)_ = 17.0), with more progressive states (B, C, D) associated with higher GCS scores (*t* = 4.13); see Table S1 for F‐statistics and p‐values of the covariates and intercept in this and other models). However, unclassifiable EEG observations, which were omitted from the above model, were significantly more likely to correspond with higher GCS scores than classifiable EEG observations (*t* = −2.59) as revealed using an LMM with ABCD classifiability (yes/no) as the EEG predictor (*p* = .010, *F*_(1,296)_ = 6.71).

ABCD type also significantly predicted the presence/absence of consciousness in patients, as inferred from GCS subscales (*p* = .0037, *F*_(1,268)_ = 8.57), with more progressive states (B, C, D) associated with consciousness (*t* = 2.93); 120 out of 302 (39.7%) EEG observations included in the GLMM corresponded to conscious states. ABCD types of B or higher were rarely observed outside of conscious states, with only 8.2% (15 out of 182) of unconscious states in the GLMM coinciding with an ABCD type of B or higher.

Next, we fit the same models substituting θα type for ABCD type. Similar to ABCD type, θα type was significantly predictive of both GCS (*p* = <.0001, *F*_(1,296)_ = 16.2) and conscious state (*p* = <.0001, *F*_(1,296)_ = 15.6), where θα + patients were more likely to have higher GCS scores (*t* = 4.03) and to be conscious (*t* = 3.95). Adding θα type as a predictor to the LMM fit using ABCD type only improved prediction of GCS scores before adjusting our α level for the additional hypotheses outlined in the introduction (*p* = .032, likelihood ratio stat = 4.59); thus, a larger sample might demonstrate an added value for including both predictors in the model. On the other hand, adding ABCD type as a predictor to the GLMM fit using θα type unambiguously improved prediction of conscious state (*p* <.0001, likelihood ratio stat = 75.4).

As a benchmark to compare the above models against, we also predicted GCS and conscious state using spectral power in the delta, theta, alpha, and beta frequency bands. Absolute alpha (*p* = .0037, *F*_(1,293)_ = 8.56) and beta (*p* = 2.9 × 10^−5^, *F*_(1,293)_ = 18.1) power significantly predicted GCS, with lower alpha power (*t* = −2.93) and higher beta power (4.25) corresponding to higher GCS scores after accounting for covariates. Note, however, that alpha power was positively related to GCS in raw correlations that did not control for other predictors (absolute power, *r* = .29; relative power, *r* = .065). Adding absolute alpha power to LMMs that predicted GCS using ABCD type (*p* = .19, log likelihood stat = 1.70) or θα type (*p* = .23, log likelihood stat = 1.41) did not significantly improve model fit in either case. However, adding absolute beta power to the LMM that predicted GCS using θα type significantly improved model fit (*p* = .0028, likelihood ratio stat = 8.93), and a trend level improvement was observed when added to the LMM that predicted GCS using ABCD type (*p* = .019, likelihood ratio stat = 5.52). Using relative power, we again found that alpha (*p* = .0022, *F*_(1,293)_ = 9.58) and beta (*p* = .0011, *F*_(1,293)_ = 10.9) power significantly related to lower and higher GCS scores, respectively. Model fits were significantly improved when relative alpha power was added to LMMs predicting GCS (ABCD type: *p* = 3.2 × 10^−4^; θα type: *p* = 5.1 × 10^−5^), but relative beta power did not significantly improve model fits (ABCD type: *p* = .079, log likelihood stat = 3.08; θα type: *p* = .32, log likelihood stat = 0.98).

Having examined spectral power as a predictor of GCS, we next examined it as a predictor of conscious state using GLMMs. No absolute power features significantly predicted conscious state, though we observed a trend for beta power (*p* = .058, *F*_(1,293)_ = 3.63), with higher power corresponding to consciousness (*t* = 1.91); however, relative delta power did significantly predict conscious state (*p* = .0069, *F*_(1,293)_ = 7.41), with lower power corresponding to consciousness (*t* = −2.72). Nonetheless, adding relative delta power to GLMMs that predicted conscious state from ABCD type or θα type did not improve model fit (the original models lacking relative delta power featured greater maximized log‐likelihoods and thus no test was performed).

Next, we used multiple linear regression with resampling to investigate whether ABCD type predicts chronic outcomes as measured with GOSe (again, types B, C, and D were grouped together to avoid outlier effects). Four patients missing GOSe score were excluded and four previously excluded patients with fewer than three EEG observations were reincluded here, see Table 2. The number of postinjury days to follow up did not correlate with GOSe scores (*r* = .05, *p* = .76) and thus was not considered to be a confound. For all hypotheses tested, the median *t*‐statistic across resamples stabilized after ~1,000 resamples (Figure S4), that is, well within the number of resamples we performed (*N* = 9,999). ABCD type did not significantly predict GOSe (*p* = .37, *t* = 0.92). We then substituted θα type for ABCD type in the original multiple linear regression model and repeated the resampling procedure. As with ABCD type, θα type did not significantly predict GOSe (*p* = .52, *t* = 0.65). Neither absolute nor relative power in any frequency band significantly predicted GOSe.

**FIGURE 4:**
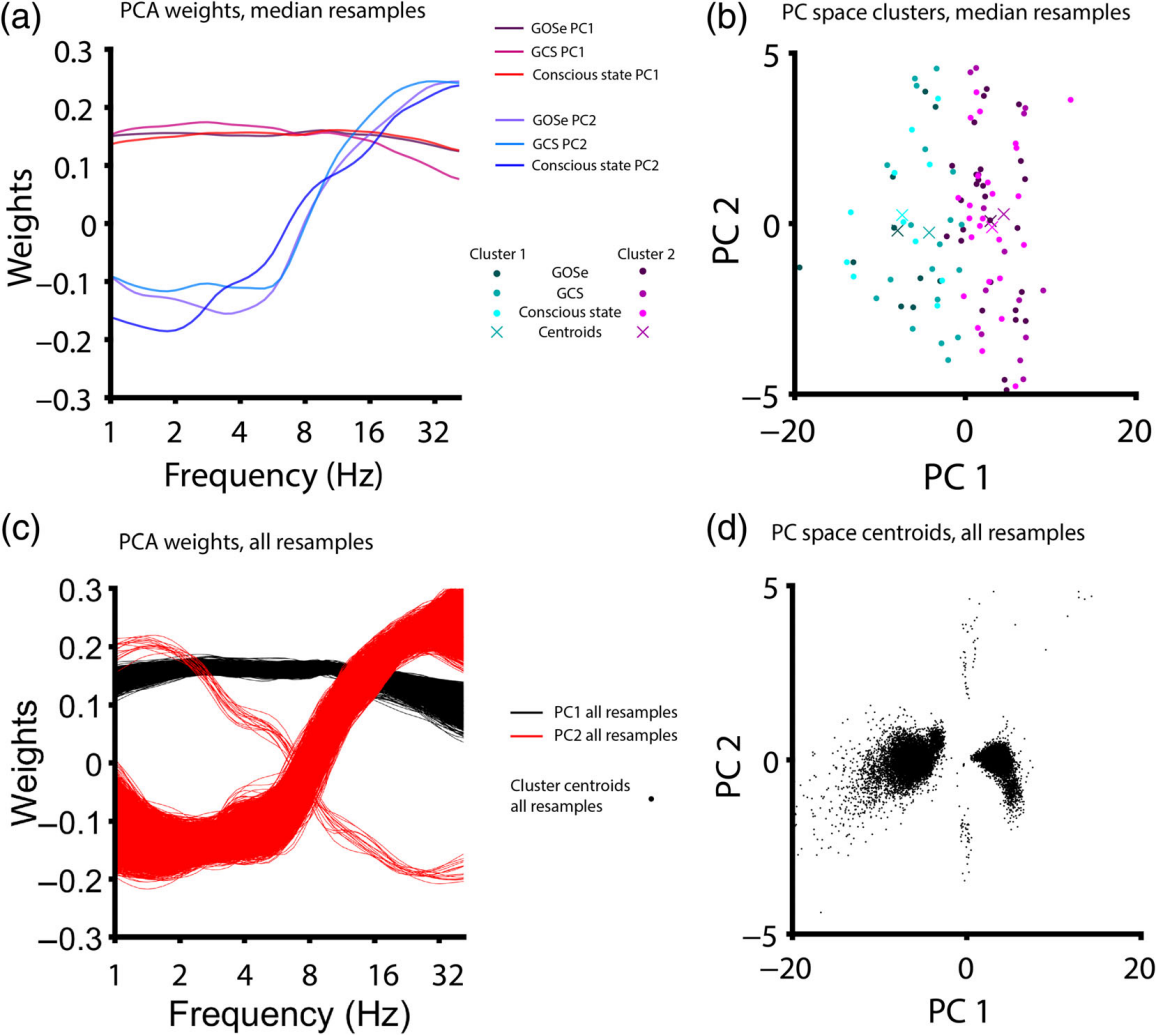
Principal component weights and clusters from resampling. Most patients (36/38, 94.7%) had multiple usable EEG observation (Table 2, mean number of EEG observations per patient = 8.4). We therefore used resampling (*N* = 9,999 resamples) to draw one EEG observation per patient for clustering in principal components (PC) space (so that clusters are not weighted more heavily toward patients with more observations). For each model, the resample yielding the median t‐statistic was selected. (a) PCA weights yielded from the median resample for each model. PCA was performed on channel‐averaged and log‐scaled EEG power spectra to cluster patients in PC space. For each model (predicting Glasgow Outcome Scale extended/GOSe, Glasgow Coma Scale/GCS or conscious state), the first PC reflected the overall EEG power (i.e., a roughly equal weighing of power across all frequencies; variance explained: 75.4%, GCS and 74.9%, GOSe) and the second PC reflected power at high frequencies (variance explained: 15.2%, both models). (b) PC space clusters and cluster centroids for each model. For all models, clusters were separated along the first PC, with the second PC accounting for most within‐cluster variance. Cluster membership was then entered as a predictor into regression models. (c) PCA weights shown for all 9,999 resamples. (d) PC space cluster centroids shown for all 9,999 resamples

Finally, we used a data‐driven clustering approach to determine whether clusters based on EEG spectral power (unnormalized) would predict GCS (*n* = 37) and/or GOSe (*n* = 33). Once again, we utilized *N* = 9,999 resamples to choose randomly one EEG observation per patient per resample. Two PCs were retained that, for all resamples, explained at least 80% of the variance in spectral power (resamples that did not explain this proportion of variance in the first two PCs were discarded and not counted toward *N*). K‐means clustering was used to identify clusters in PC space (see Figure 4). Each resample yielded one cluster whose centroid had a positive PC_1_‐coordinate and one cluster whose centroid had a negative PC_1_‐coordinate (Figure 4d), and clusters were therefore labeled accordingly. Cluster labeling did not significantly predict GCS (*p* = .11, *t* = 1.64), conscious state (*p* = .32, *t* = 1.00), or GOSe (*p* = .68, *t* = 0.42). See Tables S2–S4 for *p*‐values, *t*‐statistics, and *F*‐statistics from resampled tests.

### 3.2 Relating thalamically‐driven oscillations to mesocircuit recovery in a small sample

We identified 11 patients (seven male; age at injury: 20–59 years; 35 ± 15, mean ± *SD*) with usable fMRI data within 48 hr of at least one EEG observation, including Patient 9 (Table 2) who was excluded from prior analyses due to lack of medication data that were not covaried for in the present analysis (see Section 2). For this smaller cohort, we then examined the ABCD type of all EEG observations occurring within 48 hr of scanning. We determined that θα type was more amenable to correlational analysis than ABCD type (see Section 2), given that our samples were heavily skewed toward the A‐type, with 9 out of 11 patients showing only A‐type or unclassifiable EEG observations within this time window; by comparison, θα type displayed a weaker skew and greater variance, with the majority (6 out of 11) of patients exhibiting a mixture of both θα + and θα‐ EEG observations (Table 3). We therefore correlated the proportion of channels with a θα peak (peak proportion), averaged across EEG observations within 48 hr of scanning, with fMRI connectivity.

**TABLE 3:** EEG observations included in correlations with fMRI connectivity

ID	Age at injury (years)	GCS	Sex	Modal ABCD‐type	Modal θα type	Type proportion	Peak proportion	EEG time post‐fMRI (hr)
2	20	7	Female	A	FALSE	0.077	0.077	7.3
2	20	4	Female	A	FALSE	0.385	0.462	−12.7
2	20	7	Female	A	FALSE	0.000	0.000	19.3
6	22	10	Male	Unclassifiable	TRUE	0.000	1.000	−9.6
8	23	5	Female	A	FALSE	0.000	0.000	3.6
8	23	3	Female	A	FALSE	0.000	0.000	15.6
8	23	3	Female	A	FALSE	0.000	0.000	−16.5
8	23	3	Female	A	FALSE	0.000	0.000	27.6
8	23	3	Female	A	FALSE	0.000	0.000	−28.5
8	23	6	Female	Unclassifiable	TRUE	0.000	1.000	−40.5
8	23	4	Female	A	FALSE	0.000	0.000	43.3
9	23	3	Female	A	FALSE	0.000	0.000	34.1
10	25	3	Male	A	FALSE	0.000	0.231	8.0
10	25	3	Male	A	FALSE	0.000	0.077	−9.8
10	25	3	Male	A	FALSE	0.000	0.000	20.2
10	25	3	Male	Unclassifiable	TRUE	0.000	1.000	−21.8
10	25	3	Male	A	FALSE	0.000	0.000	34.2
14	31	3	Male	A	FALSE	0.000	0.000	4.6
14	31	3	Male	A	FALSE	0.000	0.000	−7.4
14	31	3	Male	A	FALSE	0.000	0.000	16.6
14	31	3	Male	A	FALSE	0.000	0.000	−35.4
14	31	3	Male	A	FALSE	0.000	0.000	42.6
14	31	3	Male	A	FALSE	0.231	0.231	−47.4
18	33	6	Male	A	FALSE	0.000	0.462	−2.0
18	33	6	Male	A	TRUE	0.000	0.769	−14.0
18	33	6	Male	A	FALSE	0.000	0.000	14.0
18	33	6	Male	A	FALSE	0.000	0.000	28.0
18	33	4	Male	A	FALSE	0.000	0.000	40.0
21	39	6	Male	B	TRUE	0.077	1.000	−8.9
21	39	6	Male	B	FALSE	0.462	0.462	19.1
21	39	6	Male	B	TRUE	0.154	1.000	−20.9
21	39	7	Male	A	TRUE	0.000	0.846	−36.9
30	55	5	Female	A	FALSE	0.000	0.000	6.3
30	55	5	Female	A	FALSE	0.000	0.000	20.3
30	55	8	Female	A	FALSE	0.000	0.000	36.3
31	57	7	Male	A	FALSE	0.000	0.000	6.9
31	57	7	Male	B	TRUE	0.615	0.769	−11.6
31	57	8	Male	A	FALSE	0.000	0.000	18.9
31	57	10	Male	A	FALSE	0.000	0.154	34.9
33	59	6	Male	A	TRUE	0.000	0.846	2.7
33	59	5	Male	A	TRUE	0.000	0.846	−10.3
33	59	7	Male	A	FALSE	0.000	0.077	21.7
33	59	6	Male	A	TRUE	0.000	0.846	−22.3
33	59	7	Male	A	FALSE	0.077	0.308	37.2

*Note*: Forty‐four EEG observations from 11 patients were included in the correlation of EEG with fMRI connectivity measures. EEG observations were considered if they fell within 48 hr of MRI. Type proportion gives the proportion of EEG channels with a type progressed beyond the A‐type (B, C, or D). Peak proportion gives the proportion of EEG channels with a θα peak. EEG time post‐fMRI gives the number of hours before that the EEG observation took place after the patient's MRI scan (negative values indicate that the EEG observation preceded the MRI scan). Given the more favorable statistics of peak proportion (less skew and greater variance), this measure was correlated with fMRI connectivity (Figure 5) after averaging across all available observations.

**FIGURE 5:**
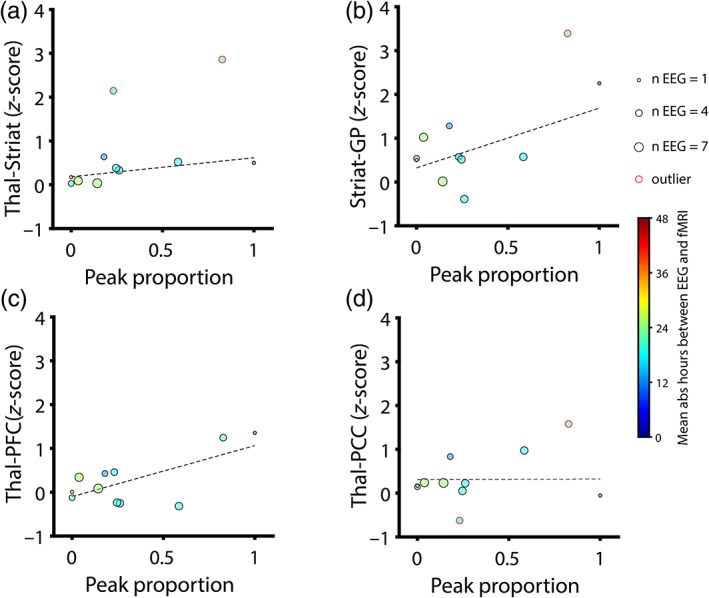
Correlations of EEG (proportion of channels with θα peak or “peak proportion”) with fMRI connectivity. Peak proportion was averaged across all usable EEG observations within 48 hr of MRI. Data points are sized proportionally to the number of averaged EEG observations and colored according to the mean hours elapsed between EEG and MRI (absolute value, warmer colors indicate greater mean time elapsed). Outliers (>3 scaled median absolute deviations from the median of either variable) are indicted with red circles around data points and were excluded from analysis. Dotted black lines represent the least‐squares linear fit for included data points. We corrected for testing across four region of interest (ROI) pairings using false discovery rates (FDR). Correlations are reported as Pearson coefficients. (a) Peak proportion versus *z*‐scored thalamo‐striatal connectivity: *r* = .63, *p* = .10, FDR corrected (uncorrected: *p* = .07). Note the presence of two excluded outliers. (b) Peak proportion versus *z*‐scored striatal‐pallidal connectivity: *r* = .59, *p* = .10, FDR corrected (uncorrected: *p* = .07). Note the presence of one excluded outlier. (c) Peak proportion versus *z*‐scored thalamo‐prefrontal cortical (PFC) connectivity: *r* = .68, *p* = .08, FDR corrected (uncorrected: *p* = .02). (d) Peak proportion versus *z*‐scored thalamo‐posterior cingulate cortical (PCC) connectivity: *r* = .01, *p* = .97, FDR corrected (uncorrected: *p* = .97). Note the presence of two excluded outliers

We found a trend relating peak proportion to fMRI connectivity between thalamus and PFC (*r* = .68, Pearson coefficient, *p* = .08, FDR corrected). Trend‐level relationships were also observed between peak proportion and thalamo‐striatal (*r* = .63, *p* = .10, FDR corrected) and striatal‐pallidal BOLD signal coupling (*r* = .59, *p* = .10, FDR corrected). No relationship was observed between peak proportion and BOLD signal coupling between thalamus and PCC (*r* = .01, *p* = .97, FDR corrected). See Figure 5 for scatter plots and correlations. To ensure that these correlations were not overly sensitive to the 48‐hr time limit used to select EEG observations, we also performed a sensitivity analysis and computed Pearson coefficients for time windows ranging 6–72 hr in length. Correlations appeared stable and maximized usable data when EEG observations were included within 13–51 hr of fMRI scanning (Figure S5). Thus, our 48‐hr time limit appears to maximize the amount of available data without including EEG observations outside of the observed window of stability.

## 4 DISCUSSION

Here, we have shown that EEG oscillations emerge as patients recover consciousness following TBI. Our findings build on previous work describing the ABCD model in largely anoxic patients by demonstrating that the model correlates with behavioral responsiveness (as measured by the total score of the GCS indicating recovery from TBI) and consciousness (as measured by specific scores on the subscales of the GCS) in TBI and outperforms categorization based on data‐driven clusters. When considering spectral power features that also predict these variables, only relative alpha power significantly improves model fit when added as a predictor alongside ABCD type. Given its ability to predict conscious state, the ABCD model may have applications as a diagnostic biomarker, for example, to detect instances of covert consciousness (Huang et al., 2018; Monti et al., 2010) in patients lacking behavioral responsiveness. However, our findings suggest that, in acute patients, more progressive types (C and D) are rare, as patients may generally achieve this level of recovery after leaving the ICU. Accordingly, a more parsimonious categorization of EEG type based on the presence or absence of θα (4–12 Hz) peaks may be equally useful, as it concentrates specifically on thalamically‐entrained cortical rhythms that form the core of the ABCD model (see Table 1). Furthermore, this approach has several practical advantages over the ABCD model: (a) all usable data are classifiable, (b) one classification will capture the majority of channels for all EEGs with an odd number of channels, and (c) because all data are classifiable, this approach does not introduce the sampling bias that occurs using the ABCD model. Finally, our study is the first to offer provisional evidence of the relationship between EEG and mesocircuit recovery, as measured with fMRI connectivity. Importantly, as predicted by the mesocircuit hypothesis, which emphasizes central thalamic projections to frontal cortex (Schiff, 2016), we found a stronger relationship between θα peak proportion and coupling between thalamus and PFC, versus coupling between thalamus and PCC.

### 4.1 EEG oscillations as a readout of mesocircuit recovery

The mesocircuit model explains postinjury forebrain dysfunction in terms of inactive striatal medium spiny neurons (MSNs; Schiff, 2010, 2016). MSN firing rates may be particularly sensitive to diffuse injury, as they require high levels of both dopaminergic neuromodulation and spontaneous background corticostriatal and thalamostriatal synaptic input to reach firing threshold (Grillner, Hellgren, Menard, Saitoh, & Wikström, 2005). When these necessary conditions are disrupted by diffuse, multifocal brain‐injury, GABAergic MSNs go offline, and the GPi is released from striatal inhibition (Schiff, 2010). As a result, GPi powerfully inhibits central thalamus, leading to functional deafferentation of cortex (Figure S6). Furthermore, the striatum receives weaker cortical and thalamic drive as a result of GPi inhibiting thalamus, leading to a positive feedback loop in which GPi is further disinhibited and central thalamus further inhibited.

As originally proposed by Schiff, the ABCD model is a readout of mesocircuit recovery, with thalamically‐driven EEG oscillations indicating progressive levels of thalamocortical integrity (Schiff, 2016). Consistent with this hypothesis, we found that ABCD type predicts conscious state, suggesting clinical application as a diagnostic biomarker in DOC. Because frontal cortical areas receive denser projections from central thalamus than other cortical areas (Deschenes, Bourassa, & Parent, 1996; Morel, Liu, Wannier, Jeanmonod, & Rouiller, 2005), frontal cortex is proposed to be disproportionately affected by mesocircuit dysfunction (Schiff, 2010, 2016). Accordingly, we found that the proportion of EEG channels displaying a θα peak is correlated with thalamo‐prefrontal connectivity (*r* = .68), but not with thalamo‐posterior cingulate connectivity (*r* = .01), in a small subsample of patients. Although neither relationship was significant after correcting for multiple comparisons, the former correlation appears promising and therefore warrants further investigation in larger samples. Furthermore, we also observed provisional, trend‐level evidence relating EEG peak proportion to subcortical mesocircuit connections (thalamo‐striatal and striatal‐pallidal connectivity). Taken together, our findings in this small subset of patients are a promising, albeit inconclusive, indicator that EEG oscillations in the 4–12 Hz range may be reflective of mesocircuit recovery.

### 4.2 Improving the ABCD model

While alternative models that predicted acute variables based on spectral power—used as benchmarks for the ABCD model—found that both absolute and relative alpha and beta power predicted GCS and that relative delta power predicted conscious state, only relative alpha power significantly improved the fit of any model with ABCD type as a predictor, as determined using log likelihood ratio tests. Adding absolute beta power, rather than relative alpha power, to the same model resulted in a trend‐level improvement, but significantly improved model fit in the same LMM with θα type substituted for ABCD type. These results suggest that incorporating both information regarding peaks in frequency bands and the area under the curve in one or more frequency bands might improve the utility of the ABCD model as a diagnostic biomarker in DOC. Surprisingly, we found that even though the presence of alpha peaks in the ABCD model was associated with higher GCS scores, alpha power itself was associated with lower GCS scores in our models, though only after accounting for beta power and other covariates, as the raw correlation between alpha power and GCS was positive for both absolute and relative power when not controlling for other predictors. This multifaceted relationship between alpha oscillations and behavioral recovery underscores the potential importance of considering both oscillatory power and peaks.

Furthermore, as the resonant frequency of alpha oscillations changes with development and aging (Chiang, Rennie, Robinson, Van Albada, & Kerr, 2011; Donoghue et al., 2020), the area under the curve in the alpha frequency range might be more sensitive to alpha activity than the presence or absence of a local maximum in the alpha range. For example, consider a slow 7 Hz posterior alpha rhythm peak that would still “leak” energy into the flanking 8–12 Hz band. Additionally, alpha and other oscillations could perhaps be more reliably detected in the ABCD model using the FOOOF (fitting oscillations and one‐over f) algorithm recently introduced by Donoghue et al. (2020) for identifying EEG oscillations in a data‐driven manner.

Finally, given that delta oscillations are widely regarded as indicators of cortical down states and unconsciousness (Buzsaki, 2006; Koch, Massimini, Boly, & Tononi, 2016; Massimini, Ferrarelli, Sarasso, & Tononi, 2012), one might be surprised by our finding that relative delta power, while predictive of conscious state, did not improve the fit of any peak‐based model, especially given that these models did not already consider any peaks <4 Hz. While delta oscillations are clearly seen in states of unconsciousness including the slow wave sleep (Brown, Lydic, & Schiff, 2010; Franks, 2008; Murphy et al., 2011), anesthesia (Franks, 2008; Murphy et al., 2011; Purdon et al., 2013; Supp, Siegel, Hipp, & Engel, 2011), and DOC (Hussain et al., 2019; Kaplan, 2004; Sutter & Kaplan, 2012) including coma and the vegetative state (Sutter & Kaplan, 2012), high amplitude delta oscillations can nonetheless be observed in a variety of circumstances in which individuals are fully conscious and responsive, such as Angelman syndrome (Frohlich et al., 2020); for a comprehensive review of this and other cases, see Frohlich, Toker, and Monti (2021). Based on these other findings and our results herein, the consideration of parameters in the delta band may not be necessary for improving the ABCD model.

Although the ABCD model outperformed a data‐driven clustering approach based on PCA and K‐means clustering, it is possible that further refinement of the clustering model or a more sophisticated unsupervised learning approach could yield competitive results. Furthermore, while the clustering approach assigned all EEG observations to a cluster, the ABCD model was unable to classify some EEG observations, resulting in a sampling bias revealed in our study using an LMM, where EEG observations corresponding to higher GCS scores were less likely to be classified. This is likely due to the fact that in conscious states, there are a greater number of “illegal” peak combinations that cannot be classified (e.g., a beta peak without any accompanying theta or alpha peak). This bias can be addressed by replacing ABCD type with θα type, which allows all EEG observations to be classified.

We also observed that dynamic regimes from the ABCD model reflecting high levels of thalamocortical integrity (C and D types) were rare in acute patients. This is perhaps unsurprising, as more recovered patients would exhibit C and D types, but they are typically moved from the ICU to less intense care and, thus, not part of our acute sample. We must therefore consider whether circuit‐level recovery precedes behavioral recovery early enough to be useful for predicting outcomes, as an ideal biomarker should predict a patient's pending recovery before the patient is moved out of the ICU. It is possible that the mere reemergence of theta oscillations (B‐type), prior to that of other oscillations, already heralds recovery. However, we did not observe a predictive relationship between ABCD type (after grouping types B, C, and D together to avoid outlier effects) and chronic outcomes as measured with GOSe. This might be due to the differing sensitivities of the GCS and GOSe assessments, where the former is better suited for detecting the presence/absence of consciousness, whereas the latter is better suited for detecting recovery of daily living skills. Because ABCD types of B or higher were rarely observed in conscious states, ABCD type relates very closely to GCS but less well to GOSe. It is possible that had our sample included more instances of C‐types and D‐types, which might relate to recovery of daily living skills, a relationship with GOSe would have also been detected.

Finally, the mean GCS score corresponding to EEG observations that were categorized as C‐type or D‐type did not exceed that of those categorized as B‐type (Table 1, Figure 2c), suggesting that C‐type and D‐type do not indicate progressive recovery beyond B‐type. However, given the small number of EEG observations that were categorized as C‐type or D‐type, it is possible that we did not have sufficient data to observe the progressive hierarchy predicted by the ABCD model. GCS ranges for A‐type (GCS = 3–15) and B‐type (GCS = 4–15) were both broad, suggesting low specificity. A‐type was observed at ceiling (GCS = 15), though conversely, B‐type was never observed at floor (GCS = 3). Given these data, EEGs that have progressed beyond A‐type likely indicate that patients have already begun recovering behavioral responsiveness.

Besides relating EEG to behavioral data, an important aim of our study was to validate an automated procedure for categorizing EEG observations according to ABCD type using quantitative objective criteria. We found that our automated EEG classification corresponded significantly with contemporaneous behavior, suggesting that the ABCD model can be implemented computationally, without the need for manual scoring. However, we found that 10.6% of EEG observations could not be classified according to the ABCD model due to peak combination that are not defined by the model. Additionally, since each EEG channel had five possible classifications (A, B, C, D, or unclassifiable), one classification did not capture the majority of EEG channels in all instances. The foregoing issues are solved using a more parsimonious classification between two types, θα‐ and θα+, with one type capturing the majority of EEG channels in all instances. We found that classification based on θα type performed equally well as ABCD classification in predicting GCS (both were strongly predictive) and GOSe (neither was predictive). The main advantage of the ABCD model was in improving prediction of conscious state when added to the GLMM with θα type as a predictor. Our findings suggest that the ABCD model performs similarly to a parsimonious and computationally simpler categorization scheme that infers thalamocortical integrity based on the presence of theta and/or alpha EEG oscillations in acute TBI patients.

### 4.3 Limitations and future directions

Our study has a number of methodological limitations: (a) Because EEG data were collected in a clinical setting, EEG channel placement was variable and the number of channels common to all patients was low. This precluded the use of EEG source localization or spatial filtering (e.g., surface Laplacian) in our analysis. Furthermore, although patients with more progressive EEG types (C and D) were largely absent from our study due to the fact that EEG data were only collected in the ICU, we believe that our findings have greater translatability since the need for diagnostic and prognostic biomarkers is most prevalent in the acute stage. (b) Despite collecting a large amount of EEG data (multiple days) from each patient, our patient sample size was relatively small (38 patients analyzed total, 33 patients in the main analysis), and so caution should be used in generalizing these findings to larger patient populations. (c) Patients were administered a large number of different medications, the influences of which could not be entirely controlled in our study. However, by utilizing logistic PCA, we covaried for two PCs that explained roughly two thirds of the variance in medication data. (d) The GOSe, our measure of chronic (~6 month) outcome, was conducted in some cases as a phone interview and thus an indirect assessment. The indirect nature of the assessment may have added substantial noise that could reduce correlations with EEG measures, possibly explaining our absence of findings when relating EEG to GOSe. (e) Our k‐means clustering approach to predicting GCS did not perfectly mirror that based on EEG spectral peak classification (ABCD type or θα type), as the latter approach utilized LMMs and thus discarded four patients with insufficient longitudinal data who were included in the former approach. However, we believe that our results unambiguously show no relationship between data‐driven clusters and GCS; thus, the asymmetry in our two analyses is likely inconsequential, though we acknowledge that a more sophisticated or better developed clustering or unsupervised learning approach could perhaps yield competitive results. (f) Our correlation of EEG with fMRI measures was limited by the fact that many patients were not scanned until leaving the ICU, at which point EEG was no longer acquired. Thus, only 11 patients had at least one EEG observation within 48 hr of fMRI. We were therefore likely underpowered to detect significant relationships between neural oscillations (EEG) and mesocircuit integrity (fMRI). However, after removing outliers that would easily influence our small sample, we observed statistical trends.

Two future directions are strongly encouraged by our results. Firstly, given the paucity of C and D types found in our data from acute patients in the ICU, future work should apply our automated peak fitting approach to data from patients moved to less intensive care, for whom more progressive types (C and D) may be observed. Such an investigation might be more successful in relating ABCD type to long‐term outcome, given the potentially larger spread in ABCD type after sampling more recovered patients. This sample might also help determine if a more parsimonious approach based on θα type is indeed preferable over the ABCD model, even when patients are more likely to exhibit a large spread in ABCD types. Secondly, we advocate for further work relating the proportion of EEG channels displaying θα peaks to mesocircuit functional connectivity in larger samples. Given the large correlation we observed between EEG peak proportion and thalamo‐prefrontal connectivity (*r* = .68), only three additional patients (*n* = 14) are needed to achieve >80% statistical power. Thus, even moderately larger samples may be beneficial in detecting significant correlations. Additionally, dynamic causal modeling (Friston, Harrison, & Penny, 2003) applied to larger datasets may be useful for inferring the directionality of interactions between mesocircuit nodes, for example, to show that EEG peak proportion correlates with increased thalamic drive to cortex or decreased pallidal inhibition of central thalamus.

## 5 CONCLUSIONS

Noninvasive readouts of mesocircuit recovery are desirable for diagnosing DOC in TBI patients. Because circuit‐level recovery should precede behavioral recovery, such a readout may also be more useful than behavioral scales (e.g., GCS) in predicting which patients will recover consciousness. Our findings show that the reemergence of neural oscillations tracks recovery in acute TBI patients, as relevant EEG measures correspond strongly and significantly with behavioral responsiveness and conscious state in the ICU while also exhibiting a trend of greater spatial extent (i.e., peak proportion) with increasing thalamocortical connectivity. These results suggest that thalamically‐driven EEG oscillations may serve as diagnostic biomarkers in TBI and DOC, although the failure to detect a relationship between EEG and chronic outcome in our patient cohort may be only due to the low occurrences of C and D types in our sample, and thus, may still have prognostic value when investigated at a less acute timepoint.

## CONFLICT OF INTEREST

Joerg F. Hipp is an employee of F. Hoffmann‐La Roche Ltd. Paul M. Vespa received personal fees from Ceribell and UCB Pharma outside the submitted work. All other authors report no competing interests.

## Supporting information

k of

## Data Availability

Data are available upon reasonable request to researchers who complete a material transfer agreement with UCLA.
