# Supplementary material for: Neural oscillations track recovery of consciousness in acute traumatic brain injury patients
Source: Hum Brain Mapp. 2022 Jan 25;43(6):1804–20. doi: 10.1002/hbm.25725 (PMC8933330; doi:10.1002/hbm.25725)
Supplement: Supplementary file 1 — k of [file HBM-43-1804-s001.docx]

**Supporting Information**

**Neural oscillations track recovery of consciousness in acute traumatic brain injury patients**

Joel Frohlich^1*^, Julia S. Crone^1,2^, Micah A. Johnson^1^, Evan S. Lutkenhoff^1^, Norman M. Spivak^3^, John Dell’Italia^1^, Joerg F. Hipp^4^, Vikesh Shrestha^3^, Jesus E. Ruiz Tejeda^3^, Courtney Real^3^, Paul M. Vespa^3^, Martin M. Monti^1,3^

**Author affiliations:**

^1^Department of Psychology, University of California Los Angeles, 6513 Pritzker Hall, Los Angeles, CA, USA

^2^Vienna Cognitive Science Hub, University of Vienna, Liebiggasse 5, 1010 Vienna, Austria

^3^Deptment of Neurosurgery, UCLA Brain Injury Research Center, David Geffen School of Medicine, University of California Los Angeles, Los Angeles, CA, USA

^4^Roche Pharma Research and Early Development, Roche Innovation Center Basel, Basel, Switzerland

***Correspondence to:** Joel Frohlich

Department of Psychology, University of California Los Angeles,

6513 Pritzker Hall, Los Angeles, CA, USA

[joelfrohlich@gmail.com](mailto:joelfrohlich@gmail.com)

**Supporting Materials and Methods**

**EEG data collection**

EEG data were acquired from most patients using a Nicolet Monitor (Natus Medical, Inc., Pleasanton, CA, USA); however, data from two patients (#7 and #26, see Table 1) were acquired using systems by Moberg ICU Solutions (Moberg Research, Inc., Ambler, PA, USA). Data were de-identified and exported as European Data Format (EDF) files using Persyst software (Persyst Development Corporation, Solana Beach, CA, USA). Behavioral assessments were performed several times daily using the GCS. To analyze patients at peak arousal, we extracted 30 minutes of EEG from 13 channels common to all patients (Fig. 1A) from timepoints corresponding to high GCS scores, with EEG observations spaced a minimum of 12 hours apart. This was accomplished by sorting each patient’s GCS scores that overlapped with EEG recordings from high to low, appending the highest score to a second list, and then crawling down the first list of GCS scores to find the next timepoint that was at least 12 hours apart from any timepoint on the second list and then adding this timepoint to the second list, and so on until no additional timepoints could be added to the second list without violating the 12-hour buffer. EEG observations (30 minutes each) were then extracted according to the second list’s timepoints (Fig. 1B).

**Frequency transform**

Following preprocessing and artifact reduction, we implemented a frequency transform using log-spaced Morlet wavelets (1-45 Hz, 8 wavelets per octave; *f/σ_f_* = 8.7; *σ_f_*, spectral SD). Elements of the time-frequency representation corresponding to noisy data were discarded before averaging across power estimates from temporal windows with 75% overlap to obtain a power spectral density (PSD). PSDs were log_10_ transformed, yielding units of log_10_(*µ*V^2^/Hz). EEG observations with < 30 windows corresponding to the wavelet with the lowest frequency (1 Hz) were discarded. For purposes of peak fitting and plotting, we increased frequency resolution to 100 bins per octave using a spline interpolation.

**fMRI preprocessing**

Patients in our cohort are prone to high motion. Thus, we excluded all patients from fMRI analysis with motion parameters higher than 2 mm translation and 2 degrees of rotation. The three translation and three rotation parameters, identified with FSL MCFLIRT (Jenkinson et al., 2002), were displayed across the whole run and time-series with peaks above the threshold were identified. We also excluded all patients with framewise displacement above 0.5 mm using FSL outliers as suggested by Power et al. (2012).

In addition, we implemented a regression approach rather than censoring techniques to cope with motion-related artifacts (Power et al., 2014) because censoring would interrupt the continuous time-series, lead to unequal number of timepoints across patients, does not correct for subtler influences of motion, and has broad effects on connectivity measures (Power et al., 2015). Instead, we implemented an approach using principal component analysis (PCA) in white matter and cerebrospinal fluid to define nuisance regressors (referred to as anatomical CompCor or aCompCor). CompCor has shown to outperform other approaches (Muschelli et al., 2014; Power et al., 2018). As suggested as the optimal solution (Muschelli et al., 2014), we included the number of principal components needed to explain 50% of the variance in white matter and in cerebrospinal fluid (aCompCor50). This denoising strategy, when paired with stringent data selection, is as effective as censoring (Parkes et al., 2018). For aCompCor50, individual masks were masked with the standard white matter and ventricles mask (transferred into structural space), respectively, as well as with subcortical masks of interest using fslmaths. This way, only areas of no interest, i.e., no signal related to neuronal gray matter, were included. Next, aCompCor50 was performed with these masks and results were used together with motion parameters as nuisance regressors.

Due to severe lesions in TBI patients, we skull-stripped the anatomical data using an optimized algorithm using optiBET (Lutkenhoff et al., 2014). Oblique information were removed from the image header using 3drefit with the option *-deoblique* and reoriented into right-to-left/posterior-to-anterior/inferior-to-superior (RPI) orientation using Analysis of Functional NeuroImages (AFNI) 3dresample with the option *-orient RPI*. Structural files were segmented into white matter, gray matter, and cerebrospinal fluid using FSL FAST. Success of segmentation was verified visually for each image.

Data were trimmed using fslroi, discarding the first four timepoints to allow for magnetization stabilization. For preprocessing, we used the FSL FEAT pipeline including FSL MCFLIRT (Jenkinson et al., 2002) for motion correction, slice time correction, BET brain extraction, spatial smoothing with full-width at half-maximum (FWHM) of 3 mm, and no temporal filtering. For registration of the functional files into anatomical and standard space, a linear transformation using FLIRT with different cost functions *-leastsquare*, *-normmi,* and *-mutualinfo* (depending on the patient) was chosen since it resulted in the best outcomes and the fewest loss of patients in comparison with nonlinear methods (data not shown).

**Brain Parcellation Using Independent Component Analysis**

We ran ICA with 100 components using ICASSO randinit with 10 runs, the Infomax algorithm, and a PCA Expectation Maximization with stacked datasets, floating point precision, 1000 iterations, and two reduction steps. The data has been intensity normalized and scaled to percent signal-change. Components were visually inspected to identify the default mode network. The component was binarized to create a mask using fslmaths. Within each mask, coordinates of prefrontal cortex (PFC) and posterior cingulate cortex (PCC) were identified (Allen et al., 2014).

**Seed-based connectivity analysis**

The thalamus and striatum masks were created from the segmented subcortical areas including the right and left thalamus, and right and left caudate and putamen, respectively. These were used to extract an average time-series for the seed-based connectivity analysis using fslmeants. Two seed-based connectivity analyses were performed (thalamus and striatum) using FEAT including prewhitening, FLAME 1, a confound EV text file including the six motion parameters and aCompCor50 results, as well as a custom basic waveform shape (i.e., the thalamus and striatum, respectively). In a next step, the z-values were extracted from the two resulting maps (i.e., globus pallidus for the striatum analysis and striatum, PFC, and PCC for the thalamus analysis).

**Patient medications and logistic principal component analysis**

Patients in our study were administered a very large number of medications in the intensive care unit. To appropriately account for medications, we first categorized medications from each EEG observation into one of five classes: propofol, opioids, benzodiazepines, barbiturates, and dissociatives (i.e., ketamine and dexmedetomidine). Next, we applied logistic principal component analysis (logistic PCA) to the resultant matrix of observations x medication classes to reduce the medication data to a 2-dimensional space; note that multiple observations per patient were included. This technique is a variant of PCA that is appropriate for binary variables (Landgraf, 2016; Landgraf & Lee, 2020). Logistic PCA was implemented using the logisticPCA R package (Landgraf, 2016; Landgraf & Lee, 2020). This implementation differs from older implementations of logistic PCA in that it does not require matrix factorization. Each row of the medication data matrix described medications for the patient at the time of an EEG observation. The matrix (observations x medications) was preprocessed by first removing rows corresponding to EEG observations with < 30 windows corresponding to the wavelet with the lowest frequency (1 Hz). Next, we discarded all rows corresponding to a patient missing medication data. The resulting matrix contained 308 rows of medication observations from 37 patients and 5 columns corresponding to each medication category: propofol, opioids, benzodiazepines, barbiturates, and dissociatives. Cross validation was used to select a value for the saturated model parameter, m = 6. Logistic PCA returned two PCs explaining 66.9% of the variance in medication data (Fig. S2).

**Supporting Results**

To choose one EEG observation per patient for k-means clustering and regression models relating acute EEG data to chronic outcome, we resampled data 9999 times using one EEG observation per patient. In our main analysis, we reported the p-value from the test with the median t-statistic for the EEG variable. Choosing the median resample based on the t-statistic rather than the F-statistic is arguably a more reasonable approach given that the t-distribution is symmetric, while the F-distribution is asymmetric. Nonetheless, we wished to confirm that both approaches would give similar results. We thus performed F-tests for the EEG term of each model generated by each resample and selected the median model based on the F-statistics. As in the main manuscript, the median models did not yield statistically significant results for any EEG term. The results of this analysis therefore did not depend on whether F-statistics or t-statistics were used to select median models.

**Supporting references**

Allen, E. A., Damaraju, E., Plis, S. M., Erhardt, E. B., Eichele, T., & Calhoun, V. D. (2014). Tracking whole-brain connectivity dynamics in the resting state. *Cerebral Cortex*, *24*(3), 663–676.

Jenkinson, M., Bannister, P., Brady, M., & Smith, S. (2002). Improved optimization for the robust and accurate linear registration and motion correction of brain images. *Neuroimage*, *17*(2), 825–841.

Landgraf, A. J. (2016). *LogisticPCA*. https://cran.r-project.org/web/packages/logisticPCA/

Landgraf, A. J., & Lee, Y. (2020). Dimensionality reduction for binary data through the projection of natural parameters. *Journal of Multivariate Analysis*, *180*, 104668.

Lutkenhoff, E. S., Rosenberg, M., Chiang, J., Zhang, K., Pickard, J. D., Owen, A. M., & Monti, M. M. (2014). Optimized brain extraction for pathological brains (optiBET). *PloS One*, *9*(12), e115551.

Muschelli, J., Nebel, M. B., Caffo, B. S., Barber, A. D., Pekar, J. J., & Mostofsky, S. H. (2014). Reduction of motion-related artifacts in resting state fMRI using aCompCor. *Neuroimage*, *96*, 22–35.

Parkes, L., Fulcher, B., Yücel, M., & Fornito, A. (2018). An evaluation of the efficacy, reliability, and sensitivity of motion correction strategies for resting-state functional MRI. *Neuroimage*, *171*, 415–436.

Power, J. D., Barnes, K. A., Snyder, A. Z., Schlaggar, B. L., & Petersen, S. E. (2012). Spurious but systematic correlations in functional connectivity MRI networks arise from subject motion. *Neuroimage*, *59*(3), 2142–2154.

Power, J. D., Mitra, A., Laumann, T. O., Snyder, A. Z., Schlaggar, B. L., & Petersen, S. E. (2014). Methods to detect, characterize, and remove motion artifact in resting state fMRI. *Neuroimage*, *84*, 320–341.

Power, J. D., Plitt, M., Gotts, S. J., Kundu, P., Voon, V., Bandettini, P. A., & Martin, A. (2018). Ridding fMRI data of motion-related influences: Removal of signals with distinct spatial and physical bases in multiecho data. *Proceedings of the National Academy of Sciences*, *115*(9), E2105–E2114.

Power, J. D., Schlaggar, B. L., & Petersen, S. E. (2015). Recent progress and outstanding issues in motion correction in resting state fMRI. *Neuroimage*, *105*, 536–551.

**Supporting Figures**

**
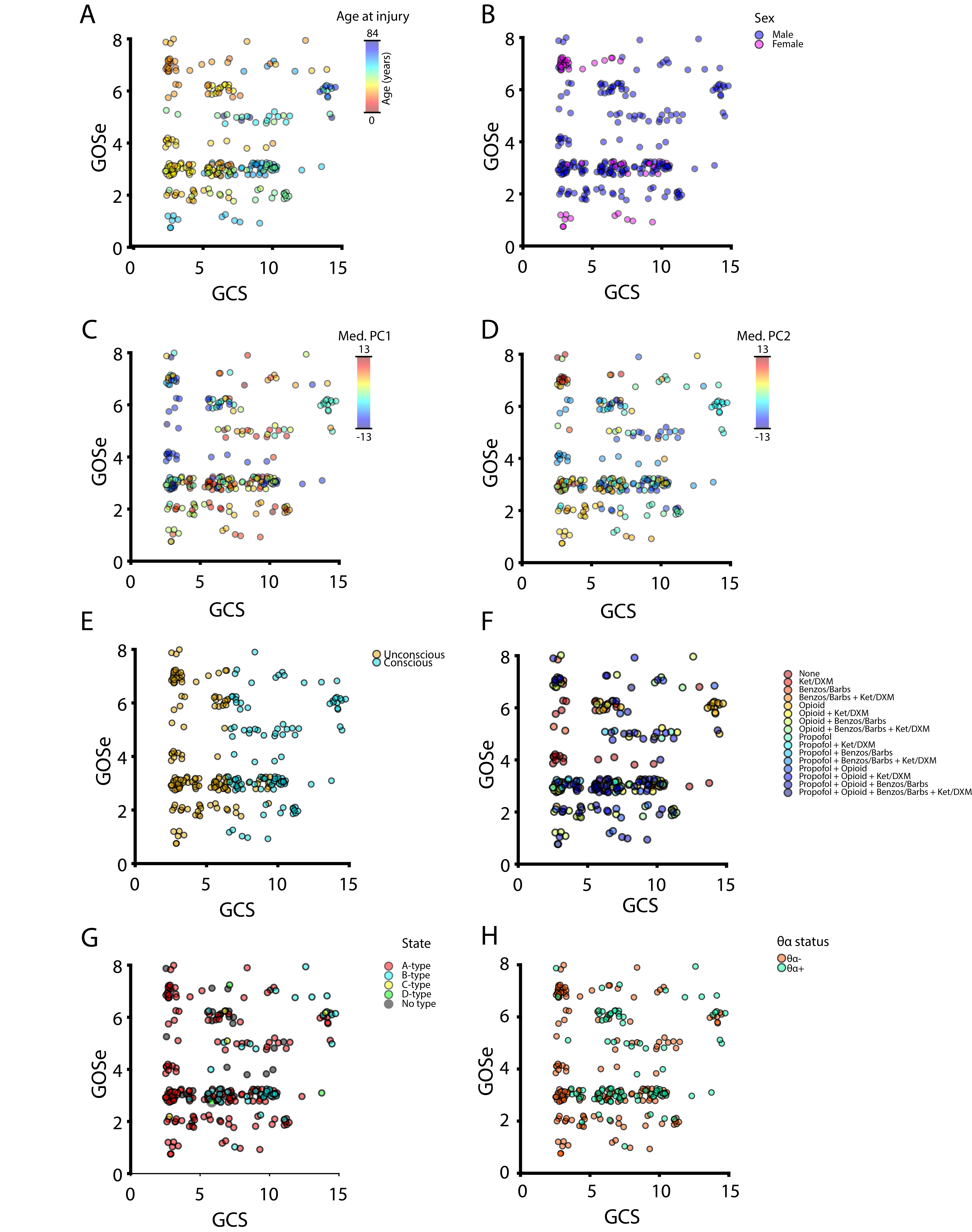
**

**Supporting Figure 1 Characteristics of EEG observations by patients’ Glasgow Coma Scale (GCS) and Glasgow Outcome Scale extended (GOSe) scores.** For display purposes, random jitter was added to scores to avoid overlap of datapoints. Note that each datapoint represents a single EEG observation, i.e., patients have multiple datapoints; four patients missing GOSe data were omitted from plots (see Table 2). Patient 8 is excluded from C, D, and F due to missing medication data (Table 2). (A) EEG observations color-coded by patients’ age at injury: warmer colors represent younger ages and cooler colors represent older ages. (B) EEG observations color-coded by sex. Our sample was heavily male dominate, reflecting high male risk in traumatic brain injury (TBI). Six out of seven female patients are depicted here; the remaining patient (Patient 30, Table 2) did not have a chronic GOSe score and was thus omitted from the figure. (C) EEG observations color-coded by the first principal component of medications obtained by logistic principal component analysis (logistic PCA). Scores from this PC relate strongly to barbiturates (see Fig. S2). (D) EEG observations color-coded by the second PC of medications obtained by logistic PCA. Scores from this PC relate strongly to dissociatives (see Fig. S2). (E) EEG observations color-coded by conscious state (conscious or unconscious, as determined by GCS subscales). (F) EEG observations color-coded by medication data: each unique combination of medication categories is a different color label, e.g., combinations involving propofol are shades of blue. (G) EEG observations color-coded by ABCD type. Note that the A-type (red) dominates low GCS observations, even for patients with good chronic outcomes (high GOSe). (H) EEG observations color-coded by θα type. Note that the θα- (orange) dominates low GCS observations, even for patients with good chronic outcomes (high GOSe).

**
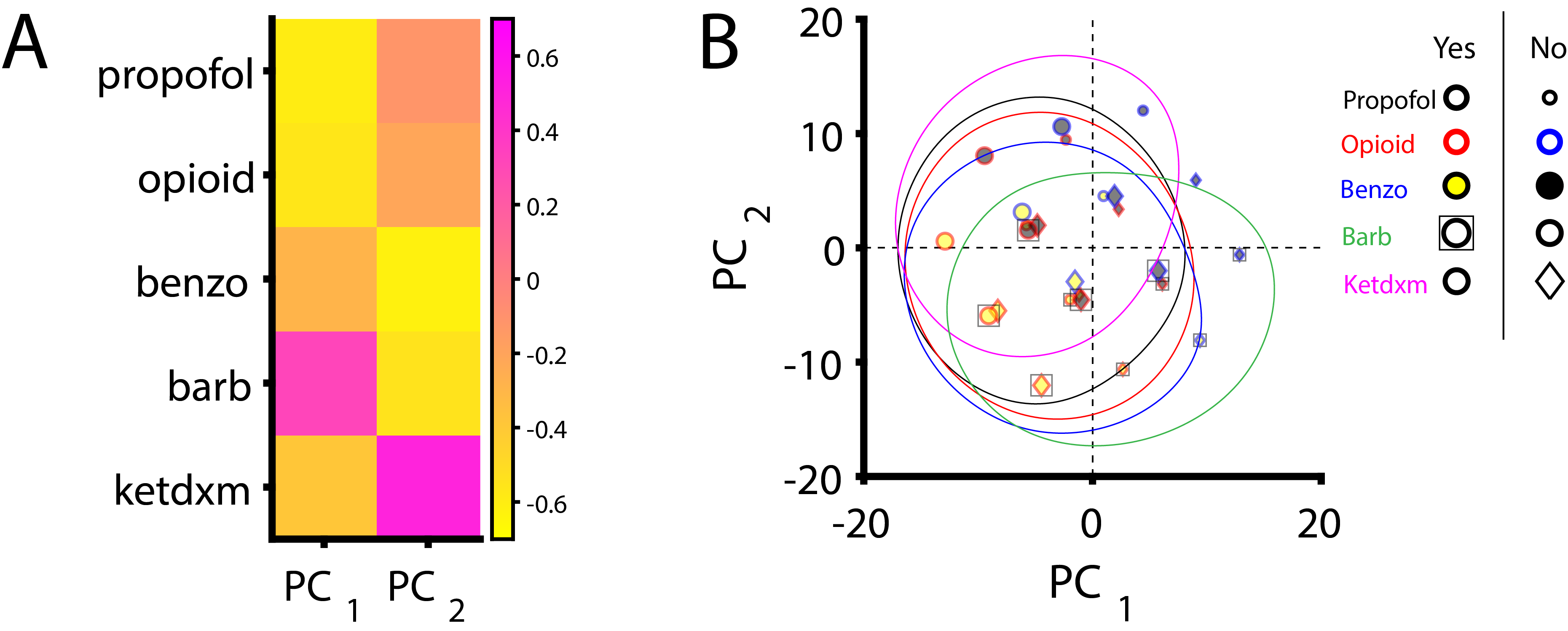
**

**Supporting Figure 2 Logistic principal component analysis weights and scores.** Patient medications were grouped into five categories and coded as binary variables: propofol, opioids, benzodiazepines (benzos), barbiturates (barbs), and dissociatives, i.e., ketamine and dexmedetomidine (ketdxm). To reduce the dimensionality of medication data, logistic principal component analysis (logistic PCA) was employed to reduce the data to two dimensions explaining 66.9% of the variance. (A) Heatmap of logistic PCA weights. Note that each PC only shows one positive weight (PC_1_: barb; PC_2_: ketdxm). (B) Representation of different medication variables in PC space. Colored contours delineate the furthest extent of each medication (propofol: black, opioid: red, benzo: blue, barb: green, ketdxm: pink). Note that barb protrudes furthest along PC_1_ and ketdxm protrudes furthest along PC_2_. Scattered points show the location of medication combinations in PC space. Propofol is coded by point size (large: true, small: false); opioid is coded by outer color (red: true, blue: false); benzo is coded by inner color (yellow: true, black: false); barb is coded by the presence (true) or absence (false) of a black square around the datapoint; ketdxm is coded by the shape of the datapoint (circle: true; diamond: false). For example, the simultaneous presence of all medication categories is denoted by large circle (red on the outside, yellow on the inside) with a black square around it; the absence of any medication category is denoted by a small diamond (blue on the outside, black on the inside). The absence of any medication occurs in the upper right quadrant while the simultaneous presence of all medications occurs in the lower left quadrant.

**
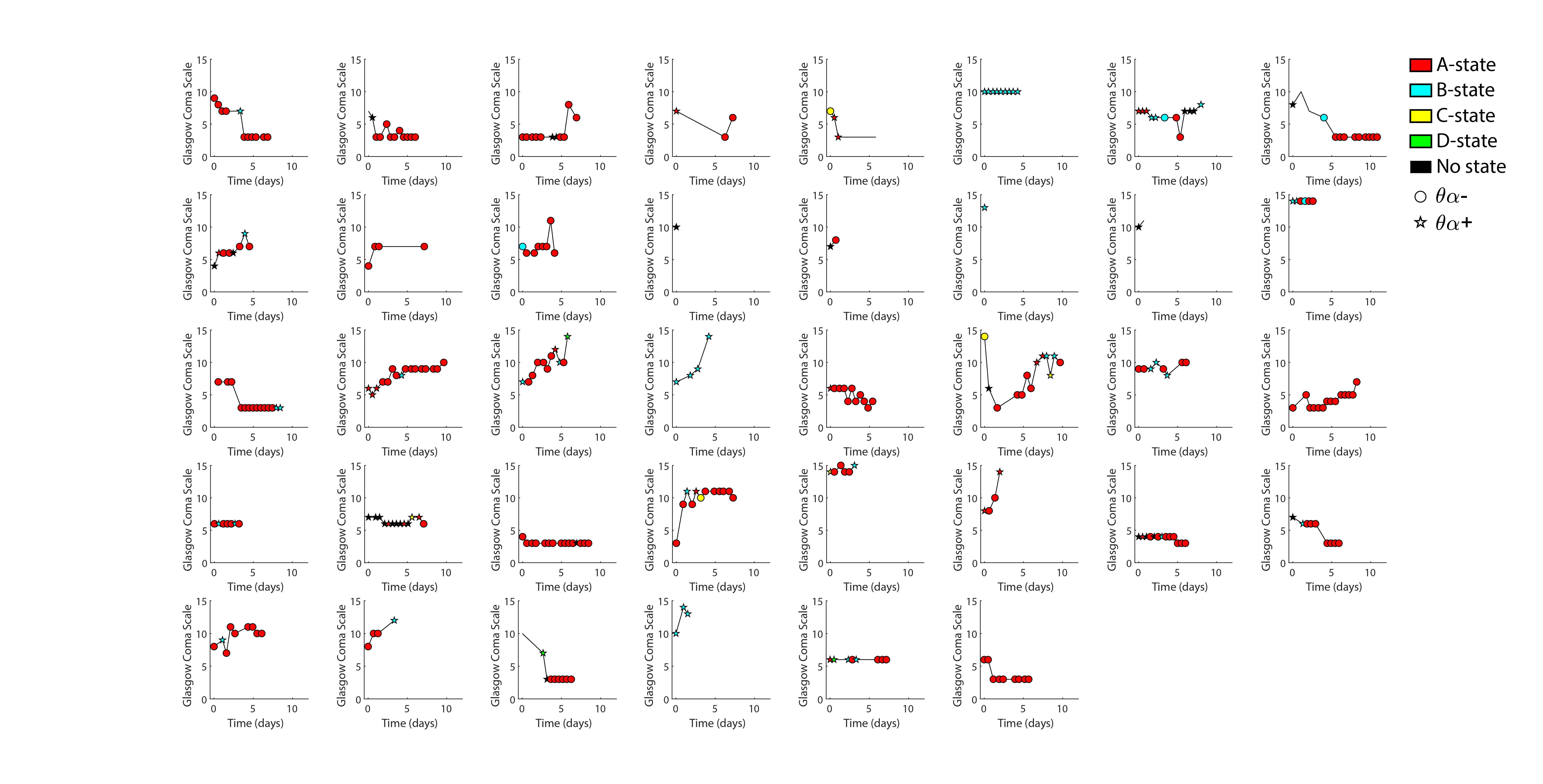
**

**Supporting Figure 3 Behavioral trajectories of all patients coded by EEG variables.** Each panel depicts data from an individual patient. ABCD type is coded by color and θα type is coded by shape. Time is referenced to the earliest time with EEG for each patient.


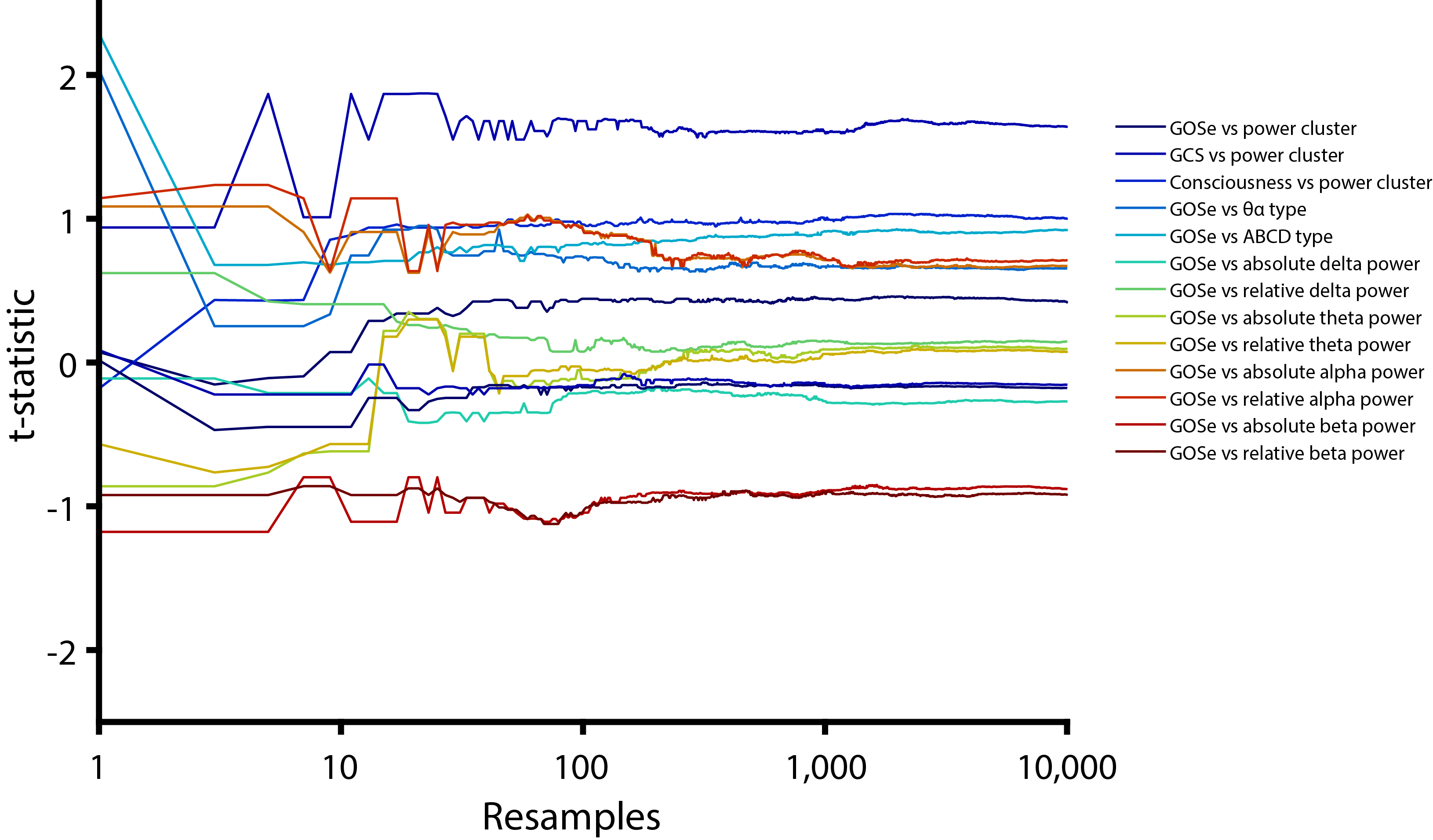


**Supporting Figure 4 Stabilization of median t-statistics as a function of number of resamples.** Note that the number of resamples is logarithmically spaced along the figure’s abscissa. Because patients had multiple EEG observations, we utilized linear mixed models (LMMs) with random effects for patients where appropriate; however, some of our hypotheses could not be addressed using LMMs. For instance, k-means clustering requires one EEG observation per patient to avoid clusters being biased toward patients with more data. Additionally, LMMs with longitudinal predictors are not valid with static outcomes, as was the case for models predicting 6-month GOSe scores (i.e., GOSe was a single timepoint for each patient, while EEG was sampled dynamically through each patient’s stay in the intensive care unit). For these analyses, we utilized a resampling procedure that randomly selected one EEG observation per patient for each of N = 9999 resamples, where N is an odd number to ensure that a middle-most sample can be selected based on t-statistics from model coefficients. Because the combinatorial space spanned by all possible combinations of EEG observations from all patients is enormous (8 x 10^31^ combinations), we wished to demonstrate that 9999 resamples would suffice for the purpose of selecting an “average” combination. For all hypotheses tested, we demonstrated that the median t-statistic (used to select an average model whose results were reported) appears to stabilize after ~1000 resamples for all hypotheses tested above. Our number of resamples is similar to that commonly used in nonparametric tests such as permutation tests (~10^4^ resamples).





**Supporting Figure 5 EEG-fMRI correlations computed for different time limits.** We adjusted the maximum allowable time between EEG observations and fMRI scanning and computed Pearson coefficients for different time limits to verify that our results were not overly sensitive to the time limit value used in our analysis (48 hours). (A) Correlations between EEG and MRI functional connectivity are roughly stable when the time limit is in the range of 13 – 51 hours, with noisy fluctuations occurring for time < 13 hours due to a limited number of patients (C) and an abrupt transition occurring between 51 and 52 hours, at which time the number of available patients drops due to outliers (C). (B) Similarly, p-values [uncorrected, visualized as -log_10_(p)] for correlations remain roughly stable within the same timeframe. The 48-hour mark (vertical dotted line) occurs late within this window of stability, thus maximizing available data (C, D) while avoiding the inclusion of EEG observations that are neither too early to capture a sufficient amount of EEG data or nor too late for EEG to reflect the level of thalamocortical integrity present during MRI scanning. Note that because outliers were excluded, the exact number of patients (C) and EEG observations (D) is different for each correlation, with the green trace showing the maximum amount of data available prior to excluding outliers.

**
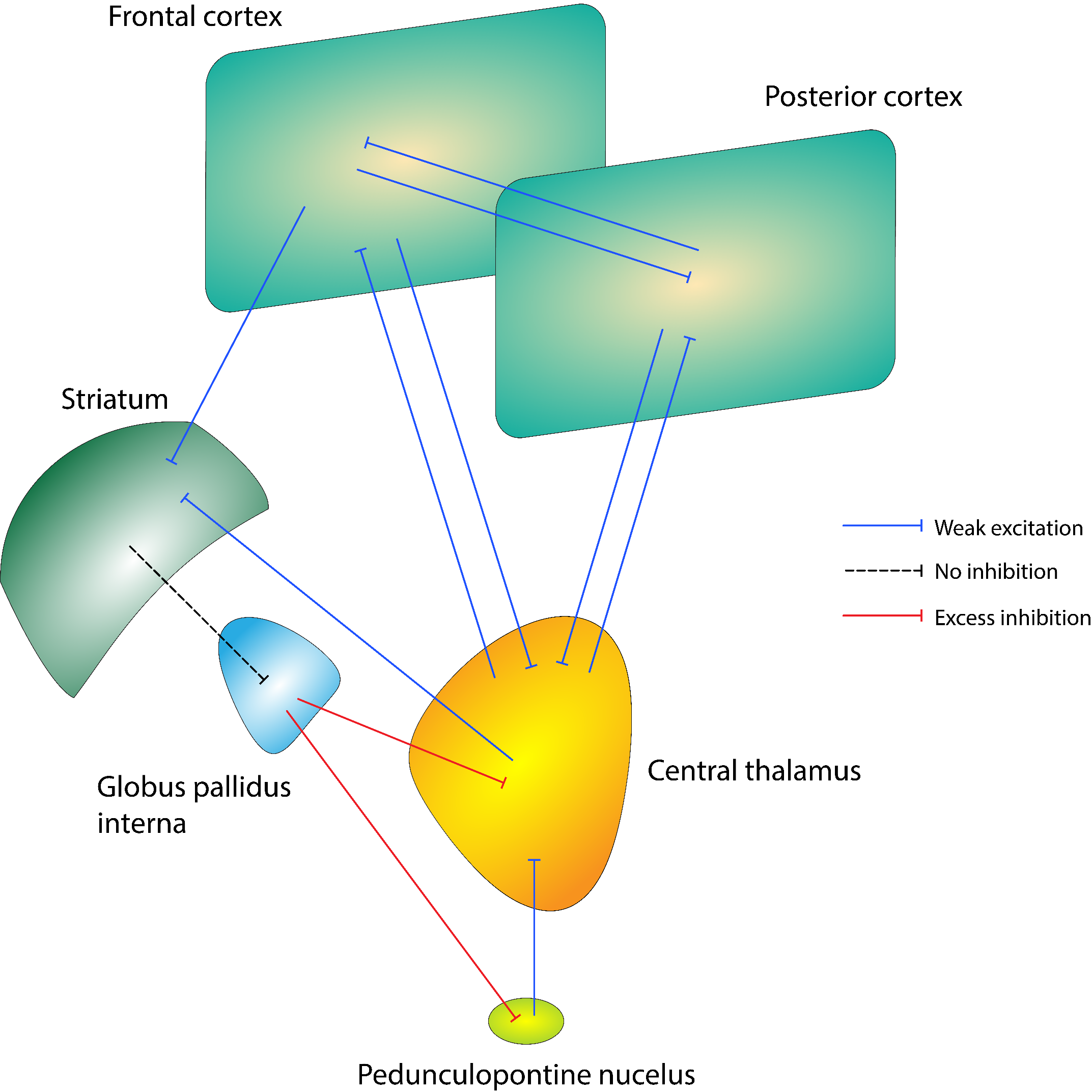
**

**Supporting Figure 6 Schematic of the mesocircuit model.** According to the mesocircuit model by Schiff, the globus pallidus interna (GPi) becomes disinhibited following diffuse brain injury, as striatal medium spiny neurons that inhibit GPi are especially vulnerable to reduced synaptic input. Excessive inhibition of the thalamus from GPi silences central thalamic nuclei, resulting in functional deafferentation of cortex and a loss or reduction of consciousness. Thus, recovery from coma or vegetative state requires restored striatal functioning and thalamocortical integrity.

| **Model** | **Term** | **df1** | **df2** | **F-stat**  **F-statistic** | **p-value** | **Significance** |
| --- | --- | --- | --- | --- | --- | --- |
| Model 1 (ABCD classifiabilitiy vs GCS) | Intercept | 1 | 296 | 12.704 | 4.25E-04 | ** |
| Model 1 | Age at injury | 1 | 296 | 11.634 | 7.37E-04 | ** |
| Model 1 | Medication PC1 | 1 | 296 | 5.013 | 0.026 | †† |
| Model 1 | Medication PC2 | 1 | 296 | 9.975 | 0.002 | * |
| Model 1 | Sex | 1 | 296 | 0.460 | 0.498 |  |
| Model 1 | Classifiable | 1 | 296 | 6.715 | 0.010 | * |
| Model 2 (θα type vs GCS) | Intercept | 1 | 296 | 8.634 | 0.004 | * |
| Model 2 | Age at injury | 1 | 296 | 11.412 | 8.27E-04 | ** |
| Model 2 | Medication PC1 | 1 | 296 | 5.380 | 0.021 | †† |
| Model 2 | Medication PC2 | 1 | 296 | 12.087 | 5.84E-04 | ** |
| Model 2 | Sex | 1 | 296 | 0.380 | 0.538 |  |
| Model 2 | θα type | 1 | 296 | 16.204 | 7.23E-05 | ** |
| Model 3 (ABCD type vs GCS, BCD grouped together) | Intercept | 1 | 268 | 8.086 | 0.005 | * |
| Model 3 | Age at injury | 1 | 268 | 11.955 | 6.34E-04 | ** |
| Model 3 | Medication PC1 | 1 | 268 | 2.959 | 0.087 | † |
| Model 3 | Medication PC2 | 1 | 268 | 7.426 | 0.007 | * |
| Model 3 | Sex | 1 | 268 | 0.325 | 0.569 |  |
| Model 3 | ABCD type | 1 | 268 | 17.020 | 4.94E-05 | ** |
| Model 4 (ABCD type vs GCS) | Intercept | 1 | 266 | 7.937 | 0.005 | * |
| Model 4 | Age at injury | 1 | 266 | 11.778 | 6.95E-04 | ** |
| Model 4 | Medication PC1 | 1 | 266 | 3.972 | 0.047 | †† |
| Model 4 | Medication PC2 | 1 | 266 | 9.753 | 0.002 | * |
| Model 4 | Sex | 1 | 266 | 0.271 | 0.603 |  |
| Model 4 | ABCD type | 3 | 266 | 8.656 | 1.68E-05 | ** |
| Model 5 (θα type vs conscious state) | Intercept | 1 | 296 | 13.733 | 2.51E-04 | ** |
| Model 5 | Age at injury | 1 | 296 | 8.488 | 0.004 | * |
| Model 5 | Medication PC1 | 1 | 296 | 3.506 | 0.062 | † |
| Model 5 | Medication PC2 | 1 | 296 | 5.044 | 0.025 | †† |
| Model 5 | Sex | 1 | 296 | 0.465 | 0.496 |  |
| Model 5 | θα type | 1 | 296 | 15.614 | 9.72E-05 | ** |
| Model 6 (ABCD type vs conscious state, BCD grouped together) | Intercept | 1 | 268 | 13.499 | 2.88E-04 | ** |
| Model 6 | Age at injury | 1 | 268 | 8.733 | 0.003 | * |
| Model 6 | Medication PC1 | 1 | 268 | 2.483 | 0.116 |  |
| Model 6 | Medication PC2 | 1 | 268 | 3.412 | 0.066 | † |
| Model 6 | Sex | 1 | 268 | 0.642 | 0.424 |  |
| Model 6 | ABCD type | 1 | 268 | 8.565 | 0.004 | * |
| Model 7 (ABCD type vs conscious state) | Intercept | 1 | 266 | 13.630 | 2.70E-04 | ** |
| Model 7 | Age at injury | 1 | 266 | 8.753 | 0.003 | * |
| Model 7 | Medication PC1 | 1 | 266 | 3.076 | 0.081 | † |
| Model 7 | Medication PC2 | 1 | 266 | 4.573 | 0.033 | †† |
| Model 7 | Sex | 1 | 266 | 0.849 | 0.358 |  |
| Model 7 | ABCD type | 3 | 266 | 3.940 | 0.009 | * |
| Model 8 (Relative power vs GCS) | Intercept | 1 | 293 | 0.000 | 0.992 |  |
| Model 8 | Age at injury | 1 | 293 | 10.600 | 0.001 | * |
| Model 8 | Relative delta power | 1 | 293 | 3.213 | 0.074 | † |
| Model 8 | Relative theta power | 1 | 293 | 2.187 | 0.140 |  |
| Model 8 | Relative alpha power | 1 | 293 | 9.582 | 0.002 | * |
| Model 8 | Relative beta power | 1 | 293 | 10.873 | 0.001 | * |
| Model 8 | Medication PC1 | 1 | 293 | 2.945 | 0.087 | † |
| Model 8 | Medication PC2 | 1 | 293 | 11.399 | 8.34E-04 | ** |
| Model 8 | Sex | 1 | 293 | 0.591 | 0.442 |  |
| Model 9 (Absolute power vs GCS) | Intercept | 1 | 293 | 3.426 | 0.065 | † |
| Model 9 | Age at injury | 1 | 293 | 10.985 | 0.001 | * |
| Model 9 | delta power | 1 | 293 | 3.174 | 0.076 | † |
| Model 9 | theta power | 1 | 293 | 0.285 | 0.594 |  |
| Model 9 | alpha power | 1 | 293 | 8.561 | 0.004 | * |
| Model 9 | beta power | 1 | 293 | 18.075 | 2.86E-05 | ** |
| Model 9 | Medication PC1 | 1 | 293 | 2.895 | 0.090 | † |
| Model 9 | Medication PC2 | 1 | 293 | 9.058 | 0.003 | * |
| Model 9 | Sex | 1 | 293 | 0.947 | 0.331 |  |
| Model 10 (Relative power vs conscious state) | Intercept | 1 | 293 | 9.354 | 0.002 | * |
| Model 10 | Age at injury | 1 | 293 | 6.584 | 0.011 | * |
| Model 10 | Relative delta power | 1 | 293 | 7.414 | 0.007 | * |
| Model 10 | Relative theta power | 1 | 293 | 0.446 | 0.505 |  |
| Model 10 | Relative alpha power | 1 | 293 | 0.656 | 0.419 |  |
| Model 10 | Relative beta power | 1 | 293 | 0.410 | 0.523 |  |
| Model 10 | Medication PC1 | 1 | 293 | 1.728 | 0.190 |  |
| Model 10 | Medication PC2 | 1 | 293 | 5.857 | 0.016 | †† |
| Model 10 | Sex | 1 | 293 | 0.311 | 0.578 |  |
| Model 11 (Absolute power vs conscious state) | Intercept | 1 | 293 | 1.901 | 0.169 |  |
| Model 11 | Age at injury | 1 | 293 | 6.850 | 0.009 | * |
| Model 11 | delta power | 1 | 293 | 0.000 | 0.991 |  |
| Model 11 | theta power | 1 | 293 | 0.009 | 0.925 |  |
| Model 11 | alpha power | 1 | 293 | 0.643 | 0.423 |  |
| Model 11 | beta power | 1 | 293 | 3.633 | 0.058 | † |
| Model 11 | Medication PC1 | 1 | 293 | 2.214 | 0.138 |  |
| Model 11 | Medication PC2 | 1 | 293 | 2.629 | 0.106 |  |
| Model 11 | Sex | 1 | 293 | 0.000 | 0.982 |  |

**Supporting Table 1 Linear mixed models and generalized linear mixed models.**

| **Test** | **EEG** | **Age at**  **injury** | **Sex** | **Medication PC1** | **Medication PC2** |
| --- | --- | --- | --- | --- | --- |
| GOSe vs power cluster | 0.678 | 0.798 | 0.117 | 0.834 | 0.239 |
| GCS vs power cluster | 0.111 | 0.022 | 0.407 | 0.904 | 0.529 |
| Consciousness vs power cluster | 0.317 | 0.071 | 0.611 | 0.971 | 0.723 |
| GOSe vs theta/alpha peak | 0.518 | 0.481 | 0.081 | 0.005 | 0.328 |
| GOSe vs A or BC state | 0.367 | 0.564 | 0.398 | 0.361 | 0.824 |
| GOSe vs absolute delta power | 0.789 | 0.590 | 0.353 | 0.286 | 0.063 |
| GOSe vs absolute theta power | 0.550 | 0.753 | 0.258 | 0.120 | 0.105 |
| GOSe vs absolute alpha power | 0.901 | 0.694 | 0.554 | 0.721 | 0.735 |
| GOSe vs absolute beta power | 0.722 | 0.829 | 0.254 | 0.399 | 0.401 |
| GOSe vs relative delta power | 0.885 | 0.679 | 0.347 | 0.443 | 0.734 |
| GOSe vs relative theta power | 0.942 | 0.999 | 0.228 | 0.379 | 0.399 |
| GOSe vs relative alpha power | 0.821 | 0.638 | 0.306 | 0.507 | 0.754 |

**Supporting Table 2 P-values from resampled tests.**

| **Test** | **EEG** | **Age at**  **injury** | **Sex** | **Medication PC1** | **Medication PC2** |
| --- | --- | --- | --- | --- | --- |
| GOSe vs power cluster | 0.420 | -0.259 | -1.620 | -0.211 | -1.205 |
| GCS vs power cluster | 1.639 | 2.412 | -0.840 | -0.122 | 0.636 |
| Consciousness vs power cluster | 1.001 | 1.803 | -0.509 | -0.037 | 0.354 |
| GOSe vs theta/alpha peak | 0.654 | 0.715 | -1.813 | 3.031 | -0.997 |
| GOSe vs A or BC state | 0.921 | -0.586 | -0.863 | 0.933 | -0.225 |
| GOSe vs absolute delta power | -0.271 | 0.545 | -0.947 | 1.091 | -1.947 |
| GOSe vs absolute theta power | 0.606 | 0.319 | -1.159 | 1.613 | -1.686 |
| GOSe vs absolute alpha power | -0.125 | -0.398 | -0.601 | 0.361 | 0.342 |
| GOSe vs absolute beta power | 0.360 | 0.218 | -1.170 | 0.858 | -0.855 |
| GOSe vs relative delta power | 0.146 | -0.419 | -0.960 | 0.780 | -0.344 |
| GOSe vs relative theta power | -0.073 | -0.002 | -1.238 | 0.896 | -0.858 |
| GOSe vs relative alpha power | 0.228 | -0.476 | -1.047 | 0.673 | 0.317 |

**Supporting Table 3 t-statistics from resampled tests.**

| **Test** | **EEG** | **Age at**  **injury** | **Sex** | **Medication PC1** | **Medication PC2** |
| --- | --- | --- | --- | --- | --- |
| GOSe vs power cluster | 0.176 | 0.067 | 2.623 | 0.045 | 1.451 |
| GCS vs power cluster | 2.686 | 5.820 | 0.705 | 0.015 | 0.405 |
| Consciousness vs power cluster | 1.001 | 3.249 | 0.259 | 0.001 | 0.125 |
| GOSe vs theta/alpha peak | 0.428 | 0.511 | 3.288 | 9.190 | 0.994 |
| GOSe vs A or BC state | 0.849 | 0.344 | 0.744 | 0.871 | 0.051 |
| GOSe vs absolute delta power | 0.073 | 0.298 | 0.897 | 1.191 | 3.792 |
| GOSe vs absolute theta power | 0.368 | 0.102 | 1.343 | 2.601 | 2.841 |
| GOSe vs absolute alpha power | 0.016 | 0.158 | 0.361 | 0.130 | 0.117 |
| GOSe vs absolute beta power | 0.130 | 0.048 | 1.368 | 0.737 | 0.731 |
| GOSe vs relative delta power | 0.021 | 0.176 | 0.922 | 0.609 | 0.118 |
| GOSe vs relative theta power | 0.005 | 0.000 | 1.532 | 0.804 | 0.736 |
| GOSe vs relative alpha power | 0.052 | 0.227 | 1.096 | 0.453 | 0.100 |

**Supporting Table 4 F-statistics from resampled tests.**
